# A Requirement of Protein Geranylgeranylation for Chemokine Receptor Signaling and Th17 Cell Function in an Animal Model of Multiple Sclerosis

**DOI:** 10.3389/fimmu.2021.641188

**Published:** 2021-03-22

**Authors:** Gregory Swan, Jia Geng, Eunchong Park, Quanquan Ding, John Zhou, Ciana Walcott, Junyi J. Zhang, Hsin-I Huang, Gianna Elena Hammer, Donghai Wang

**Affiliations:** ^1^Division of Rheumatology and Immunology, Department of Medicine, Duke University School of Medicine, Durham, NC, United States; ^2^Department of Immunology, Duke University School of Medicine, Durham, NC, United States

**Keywords:** adaptive immune response, T cells, autoimmunity, lymphocyte migration, protein geranylgeranylation

## Abstract

Precisely controlled lymphocyte migration is critically required for immune surveillance and successful immune responses. Lymphocyte migration is strictly regulated by chemokines and chemokine receptors. Here we show that protein geranylgeranylation, a form of post-translational protein lipid modification, is required for chemokine receptor-proximal signaling. Mature thymocytes deficient for protein geranylgeranylation are impaired for thymus egress. Circulating mature T cells lacking protein geranylgeranylation fail to home to secondary lymphoid organs or to transmigrate in response to chemokines *in vitro*. Mechanistically, protein geranylgeranylation modifies the γ-subunits of the heterotrimeric small GTPases that are essential for chemokine receptor signaling. In addition, protein geranylgeranylation also promotes the differentiation of IL-17-producing T helper cells while inhibiting the differentiation of Foxp3^+^ regulatory T cells. Finally, mice with T cell lineage-specific deficiency of protein geranylgeranylation are resistant to experimental autoimmune encephalomyelitis induction. This study elucidated a critical role of protein geranylgeranylation in regulating T lymphocyte migration and function.

## Introduction

T cell-mediated adaptive immune response depends on precisely controlled lymphocyte trafficking (1, 2). Under homeostatic conditions, naive T cells enter secondary lymphoid organs (SLOs) to scan for antigenic peptide on antigen presenting cells (2). In the presence of cognate antigen, antigen-specific T cells undergo activation, proliferation and differentiation into effector T cells that subsequently leave SLOs and travel through the circulation to peripheral target sites to orchestrate immune responses culminating in the elimination of pathogens or neoplastic cells (3). Likewise, such trafficking processes has also been targeted to treat autoimmune diseases.

The exquisite process of lymphocyte trafficking is critically regulated by three types of receptors-selectins, chemokine receptors and integrins (4). Selectin interaction with their ligands results in the tethering and rolling of lymphocytes along vascular wall that in turn enables the contact between chemokine ligands on endothelial cells and chemokine receptors on lymphocytes (5). The chemokine and its receptor interaction initiates a so-called “inside-out” signaling cascade within lymphocytes that converts integrins from curved, inactive conformation into an extended, active one (6). Activated integrins such as lymphocyte activation antigen-1 (LFA-1) then bind to integrin ligands such as intercellular adhesion molecule-1 (ICAM-1) on the vascular endothelial cells leading to the arrest and subsequent transmigration of lymphocytes into the paracortical region of lymph nodes (1, 5).

Chemokine receptors are G-protein coupled receptors (GPCRs) that depend on heterotrimeric small GTPases to relay signals from the plasma membrane to downstream proximal signaling components such as phosphoinositol-3-kinases to initiate signaling cascades that control lymphocyte trafficking (7). Despite the dramatic expansion of our knowledge in the past few decades, how the complex signaling network downstream of chemokine receptors fine-tunes the outcome of an immune response remains incompletely understood.

Protein geranylgeranylation is a form of post-translational lipid modification of proteins with geranylgeranyl pyrophosphate (GGPP) as a substrate and regulates a plethora of essential physiological processes (8). Protein geranylgeranylation is catalyzed by the heterodimeric protein geranylgeranyl transferase-I (GGTase-I) consisting of an α and a β subunit. Using a conditional allele of the gene encoding the β-subunit of GGTase-I (*Pggt1b*) (9), we have previously elucidated molecular mechanisms by which protein geranylgeranylation regulates innate immune signaling downstream of toll-like receptors (10) and RIG-I-like receptors (11) in myeloid cells. López-Posadas et al. (12) reported that loss of Pggt1b in T cells in *Pggt1b*^*fl*/*fl*^*CD4Cre* mice led to impaired RhoA function, increased integrin α4β7 expression and preferential localization of inflammatory CD4^+^ T cells to colon and colitis. Du et al. elucidated that Pggt1b is required for thymus egress by bridging chemokine-induced activation of Cdc42 and Pak signaling (13). Both studies relied on the *Pggt1b*^*fl*/*fl*^*CD4Cre* mouse strain in which there is a severe T lymphopenia in the periphery. In addition, the majority of mature T cells in the periphery in those mice displayed an activated phenotype. These abnormalities in T cells makes it difficult to study peripheral T cell function using *Pggt1b*^*fl*/*fl*^*CD4Cre* mice.

To study how protein geranylgeranylation regulates T cell-mediated adaptive immune response, we have generated a mouse strain in which the expression of *Pggt1b* was abrogated in mature T lymphocytes by means of a distal *Lck* promoter-driven Cre and the *Pggt1b* conditional allele. Using this mouse strain, we demonstrate that protein geranylgeranylation deficiency in T cells lead to defective adaptive immune response due to impaired T lymphocyte migration. Mechanistically, we show that this impairment is, at least in part, due to the loss of geranylgeranylation of the γ-subunits of the chemokine receptor-associated heterotrimeric small GTPases. As a result, Pggt1b-deficient naive T cells are defective in targeted trafficking to SLOs while Pggt1b-deficient effector T cells are not able to emigrate from SLOs into the circulation after primary immunization. Consequently, mice with T cell-specific deletion of Pggt1b are resistant to the induction of experimental autoimmune encephalomyelitis (EAE). We further demonstrate that in the absence of protein geranylgeranylation naive CD4^+^ T cells preferentially differentiate into induced Foxp3^+^ regulatory T cells (iTregs) over IL-17-producing T helper (Th17) cells. These findings revealed a pivotal role of protein geranylgeranylation in regulating T cell-mediated adaptive immune response.

## Materials and Methods

### Mice

*Pggt1b*^*fl*/*fl*^ mice generated as previously described (9) were crossed with *dLckCre-*transgenic mice (14) to generate the *Pggt1b*^*fl*/*fl*^*dLckCre* mouse strain. *Pggt1b*^*fl*/*fl*^*dLckCre* mice and littermate control *Pggt1b*^*fl*/*fl*^ or *Pggt1*^*fl*/+^*dLckCre* mice were used in the experiments as indicated in each figure. 2D2-TCR-transgenic strain of mice (15) was purchased from Jackson Laboratories and was crossed with *Pggt1b*^*fl*/*fl*^*dLckCre* mice to generate the 2D2- *Pggt1b*^*fl*/*fl*^*dLckCre* compound transgenic mice. Mouse strains were maintained in specific pathogen-free conditions in the animal facility at Duke University, and the animal protocols were performed in accordance with the guidelines set forth by the Institutional Animal Care and Use Committees of Duke University.

### Flow Cytometry Analysis

Lymphocytes from spleen, thymus, lymph nodes, and blood were stained with antibodies in FACS buffer that contains PBS, 2%BSA and 1 mM EDTA. Leukocytes were isolated from spinal cord of mice immunized to induce EAE according to a protocol described by Manglani et al. (16). The antibodies used in our analysis is listed here: CD4 (clone GK1.5), CD8α (clone 53–6.7), CD25 (clone PC61), CD62L (clone MEL14), TCRβ (clone H57-597), CD45(clone 30-F11), CD44 (clone IM7), CD11c (clone N418), CD11b (clone M1/70), CD64 (clone X54-5/7.1), I-A/I-E (clone M5/114.15.2), Ly6C (clone HK1.4), mIL-17A (clone TC11-18H10.1), mGM-CSF (clone MP1-22E9), IFNγ (clone XMG1.2), mIL-10 (clone JES5-16E3), mFoxp3 (FJK-16s), CCR6 (clone 29-2L17), CCR7 (clone 4B12), Integrin α4β7 (clone DATK32). Those antibodies are from Biolegend. Intracellular cytokine staining and Foxp3 staining were performed per manufacturer's instructions (Invitrogen GAS003 for cytokines and eBioscience 00-5523-00 for Foxp3). Flow cytometry data were acquired on BD FACSCanto II or BD LSRFortessa (BD Biosciences) and analyzed using Flowjo (BD). For leukocytes isolated from EAE mice described in **Figure 5** and Supplementary Figure 3, we used an antibody panel and gating strategy described by Caravagna et al. (17).

### *In vitro* T Cell Cultures

Pooled cells from spleen and lymph nodes were first enriched for CD4^+^ T cells with Magnisort mouse CD4 T cell enrichment kit (eBioscience 8804-6821-74) and then stained and sorted for CD4^+^ CD25^−^ CD44^low^CD62L^high^ naive T cells. Sorted naive CD4^+^ T cells were used for *in vitro* cultures in IMDM (Life Science 12440-053) supplemented with β-mercaptoethanol, 10% fetal bovine serum and 1% penicillin-streptomycin. For survival experiment, naive T cells were cultured in the presence or absence of mIL-7 (20 ng/ml). For T cell activation, naive T cells were plated on cell culture plates pre-coated with goat anti-hamster IgG (MP Biomedicals, 0856984) and in the presence of hamster anti-mouse CD3ε (eBioscience 16-0031-86) and hamster anti-mouse CD28 (eBioscience 16-00281-86) antibodies and differentiated into different subsets using reagents and recipes listed in the table below: mIL-2 (Biolegend 575404), mIL-12 (Biolegend, 577004), mIL-4 (Biolegend 574304), mTGFβ1 (Biolegend 736102), mIL-6 (Biolegend 575702), αIFNγ (Biolegend 505812), αmIL-4 (Biolegend 504108), αmIL-6 (Biolegend 501110), mIL-1 (Peprotech 211-11B), mIL-23 (R&D systems 1887 CF), mIL-7 (Biolegend 577806). For the culture of pathogenic Th17 cells see *EAE induction by adoptively transfer of Th17 cells*.

Final Concentrations of Differentiation Antibodies and Cytokines.

**Table:** 

	Th0	Th1	Th2	Th17	Treg
α-CD3ε	1.0 μg/ml	1.0 μg/ml	1.0 μg/ml	1.0 μg/ml	1.0 μg/ml
α-CD28	1.0 μg/ml	1.0 μg/ml	1.0 μg/ml	1.0 μg/ml	1.0 μg/ml
mIL-1				20 ng/ml (Pathogenic)	
mIL-2		20 ng/ml	20 ng/ml		20 ng/ml
mIL-4			50 ng/ml		
mIL-12		20 ng/ml			
mIL-6				50 ng/ml	
mIL-23				20 ng/ml (Pathogenic)	
mTGFβ1				0.3 ng/ml	5.0 ng/ml
α-mIL-4		0.5 μg/ml		0.5 μg/ml	0.5 μg/ml
α-mIFNγ			0.5 μg/ml	0.5 μg/ml	0.125 μg/ml
α-mIL-6					0.5 ng/ml

### Transfection of 293 Cells for the Production of Retrovirus and Infection of Th17 Cells

For preparation of retrovirus, retroviral vector DNA along with an EcoPac packaging vector were transfected into 293 cells using calcium phosphate method (18). Culture media were changed into fresh ones 24 h later. Culture supernatants were harvested, filtered through a 0.45 μm filter and centrifuged at 6,000 g overnight. The opaque virus pellet was resuspended with fresh medium and used for transduction of Th17 cells by replacing the supernatant of day 3 culture of Th17 cells with the virus preparation and spinoculate at 2,000 rpm for 1 h. The Th17 cell culture plate was then incubated at 37°C for 5 h, and the medium was replaced with Th17 culture cocktail and cultured for additional 18 h before proceeding to further experiments.

### Real Time PCR

Real time PCR was performed as described previously (11) using primers listed here:

**Table:** 

*mPggt1b*	CCTTCTGTGGCATTGCGTCA	CAACAAGGCGATCTTGAGTTG
*mGapdh*	TGGCAAAGTGGAGATTGTTGCC	AAGATGGTGATGGGCTTCCCG
*mGngt1*	CTGGAGAGAATGATGGTTTCCAAATC	ACACAGCCTCCTTTGAGTTCC
*mGngt2*	CCCACGTGATCTGATTTCCAAG	CACACAAGTGCCTTTCTCCTTG
*mGng2*	ACCGCCAGCATAGCACAAG	AGTAGGCCATCAAGTCAGCAG
*mGng3*	GCACTATGAGTATTGGTCAAGCA	GTGGGCATCACAGTATGTCATC
*mGng4*	GGCATGTCTAATAACAGCACCA	CACTGGGATGATGAGGGGG
*mGng5*	ATGTCGGGTTCTTCTAGCGTC	GGTCTGAAGGGATTCGTACTT
*mGng7*	TCAGGTACTAACAACGTCGCC	CAGTAGCCCATCAGGTCTGAC
*mGng8*	TCGCATGAAGGTGTCGCAG	CTTGTCGCGGAAGGGATTCTC
*mGng10*	GCCAGCGTGAGCGCCC	GCAGCAGGGCGTCCTTGC
*mGng11*	GTCAAGTTGCAGAGACAACAGGTATCTAAATG	GATTCCCTTTACCAGAGGATCCTC
*mGng12*	ATGTCCAGCAAGACGGCAAG	GAGGTCGGTATGCCCATCAG
*mGng13*	GTCCAAGGAGATCGACAAATGC	CCAGCACCCTCATACCTTTGA

### EAE Induction

Murine myelin oligodendrocyte glycoprotein (MOG) 35–55 peptide with a sequence as MEVGWYRSPFSRVVHLYRNGK synthesized by United Biosystems was dissolved in sterile water at 2 mg/ml. Equal volume of MOG and complete Freud's adjuvant (CFA) containing 4 mg/ml of heat-killed M. tuberculosis H37 RA (Fisher Scientific DF3114-33-8) were mixed and emulsified. Two hundred microliter of the emulsified mixture were injected subcutaneously in the flanks of mice on day 0. Pertussis toxin (200 ng/mouse) was injected intraperitoneally on day 0 and day 2. Disease was scored during daytime with the criteria: 0.5, partial tail limpness; 1, tail limpness; 1.5, reversible impaired righting reflex; 2, impaired righting reflex; 2.5, paralysis of one hind limb; 3, paralysis of both hind limbs; 3.5, paralysis of both hind limbs and one fore limb; 4, paralysis of both fore limbs and hind limbs; 5, death or body weight below 80% of day 0.

### EAE Induction by Adoptively Transfer of Th17 Cells

Purified naive CD4^+^ T cells (CD4^+^ CD25^−^ CD62L^high^CD44^low^) from 2D2 TCR transgenic mice (15) on *Pggt1b*^*fl*/*fl*^ or *Pggt1b*^*fl*/*fl*^*LckCre* background were activated as described in *In vitro T cell cultures* in the presence of mIL-6 (20 ng/ml), mTGFβ1 (0.3 ng/ml), anti-mIL-4 and anti-mIFNγ (0.5 μg/ml each) for 48 h and then changed into medium containing mIL-6, mIL-23, mIL-1β (20 ng/ml, each) and anti-mIL-4 and anti-mIFNγ (0.5 μg/ml, each) and cultured for an additional 72 h. 3 × 10^6^ cells/mouse were injected *i.v*. into C57BL/6 recipient mice on day 0. Disease score and body weight of recipient mice were monitored daily for 20 consecutive days.

### *In vitro* Chemotaxis

100 μl of serum free medium containing 350,000 cells (splenocytes for CCL21 chemotaxis or Th17 cells for CCL20 chemotaxis) were loaded into the upper chamber of a transwell insert (3421, Corning) as input. The lower chambers were pre-loaded with 600 μl of medium with or without CCL21 or CCL20 and the plate was incubated at 37°C, 5% CO_2_ for 6 h. CCL20 induced Gng13-reconstituted Th17 cell migration, vector or Gng13 transduced Pggt1b-deficient Th17 cells were performed as described above, migration efficiency was calculated on GFP-positive (transduced) cells. For CCL21 chemotaxis, input and cells that migrated into the lower chamber were stained for TCRβ, CD4 and CD8α and analyzed with flow cytometry to calculate the cell numbers of each subsets. Migration efficiency is calculated according to this formula: %Input = Number of cells migrated into the lower chamber/Number of cells of input.

### *In vivo* Homing of Purified CD4*^±^* Naive T Cells

CD4^+^ naive T cells were enriched using the Magnisort Naive T cell Enrichment kit (Thermo Fisher) and labeled with CellTrace Far Red kit (ThermoFisher, C34572) or Cell Proliferation Dye eFluor450 (Thermo Fisher, 65-0842-85). Cells from *Pggt1b*^*fl*/*fl*^*dLck-Cre* or *Pggt1b*^*fl*/*fl*^ littermate control mice were mixed at a 1:1 ratio and *i.v*. injected into wild-type recipient mice. Four hours later, spleen, blood, and peripheral lymph nodes were harvested and the frequency of CD4^+^ naive T cells homing into SLOs were calculated by dividing cell numbers recovered in each organ by the total cell numbers inoculated.

### Immunoblot

Cells were lysed with RIPA lysis buffer (25 mM Tris-HCl (pH 7.4), 150 mM NaCl, 5 mM EDTA, 0.1% SDS, 0.5% sodium deoxycholate, 1% triton X-100) supplemented with protease and phosphatase inhibitors. Protein concentrations were determined to ensure that each well was loaded with 5 μg of total proteins. Protein samples were resolved on SDS-PAGE gels and subjected by immunoblot with the following antibodies: Pggt1b (Sigma HPA030646), npRap1a (Santa Cruz sc1482), Phospho-Akt S473 (Cell Signaling, 4060), phosphor-Gsk3β Ser9 (Cell Signaling 9322), phosphor-Erk (Cell Signaling, 9101), phosphor-Stat3 Tyr705 (Cell Signaling, 9145), phosphor-Smad2 (Cell Signaling 3108), phosphor-Smad3 (Cell Signaling, 9520), and β-Actin-HRP (Sigma, A3854).

## Results

### Establishment of the *Pggt1b^*fl*/*fl*^ dLckCre* Strain of Mice

To study the function of protein geranylgeranylation in mature T lymphocytes, we have generated a mouse strain that abrogates the expression of *Pggt1b* specifically in T lymphocytes by crossing mice carrying the conditional *Pggt1b* allele (*Pggt1*^*fl*/^) (9) with a *dLckCre* (14) transgenic strain of mice. When combined with a loxP sites-flanked tdTomato (*tdTomato*^*Stopfloxed*^) (19) red fluorescent reporter and consistent with previous findings, the *dLckCre* transgene expression was first seen in post-positive selection double positive (DP) thymocytes that extended to CD4 and CD8 single positive (SP) cells in the thymus. However, the *dLckCre* transgene expression was not with 100% penetration in the thymus with a higher deletion efficiency in CD8 SP than in CD4 SP thymocytes (Supplementary Figures 1A–C). Consistently, quantitative RT-PCR analysis showed that *Pggt1b* expression was not significantly affected in DP thymocytes and was diminished but not abrogated in CD4 and CD8 SP thymocytes, indicating that Cre-mediated deletion of *Pggt1b* was incomplete in bulk CD4 and CD8 SP thymocytes in the thymus (Supplementary Figure 1D). This is further supported by immunoblot showing that Pggt1b expression was largely unaltered in total thymocytes from *Pggt1b*^*fl*/*fl*^*dLckCre* mice (Supplementary Figure 1E). Nevertheless, non-geranylgeranylated Rap1a protein were weakly detected in thymocytes from *Pggt1b*^*fl*/*fl*^ *dLckCre* mice, indicating that a fraction of such thymocytes had lost Pggt1b. This is consistent with the transcript abundance and the fluorescence reporter results. However, naive CD4^+^ T cells isolated from spleen and lymph nodes displayed a near complete deletion of *Pggt1b* both at the transcript and protein level (Supplementary Figures 1F,G). Therefore, we have established a mouse model with abolition of *Pggt1b* expression in peripheral mature naive T cells.

### Defective Thymocytes Egress in *Pggt1b^*fl*/*fl*^ dLckCre* Mice

**Figure 1:**
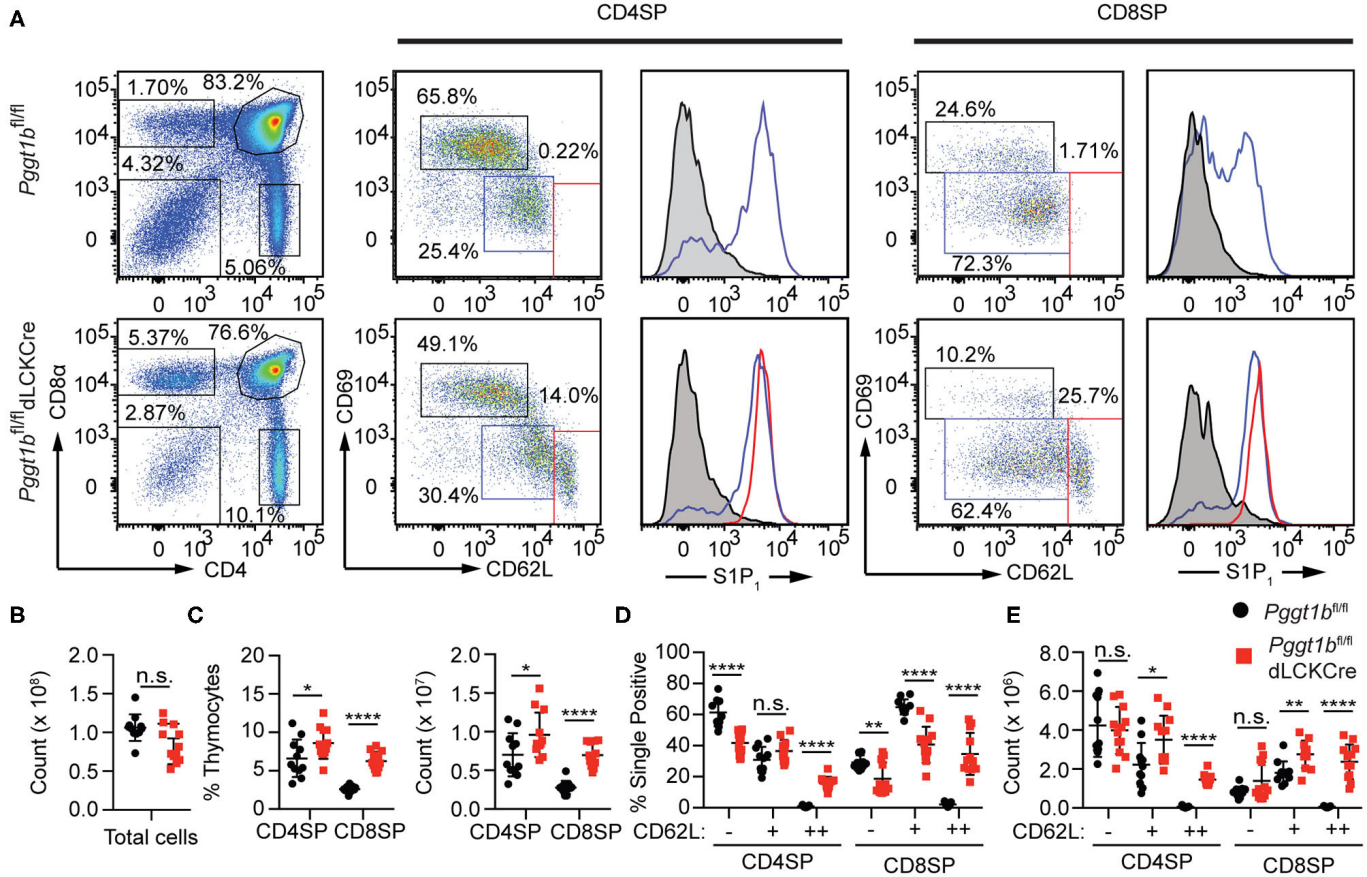
Defective mature thymocytes egress in *Pggt1b*^*fl*/*fl*^ *dLckCre* mice **(A)** Flow cytometry analysis of thymic CD4 SP and CD8 SP cell expression of CD69 and CD62L and the expression of S1P1R based on sub-populations defined by CD62L^low^ CD69^high^, CD62L, and CD69^intermediate^ or CD62L^high^ CD69^low^; **(B)** Total number of thymocytes; **(C)** the percentage and number of CD4 SP and CD8 SP of total thymocytes; **(D)** percentage and the total number of CD4 SP and CD8 SP subpopulations described in **(A)**. Each dot in the graphs **(B–E)** represents a single mouse (n.s. statistically not significant; *p < 0.05, **p < 0.01, ***p < 0.001, ****p < 0.0001, unpaired *t*-test).

Mature thymocytes that are ready to exit into the periphery are predominantly CD4 or CD8 SP, with down-regulated the activation marker CD69, and up-regulated CD62L and sphingosine-1-phosphate receptor 1 (S1PR1), the primary chemokine receptor essential for thymic egress (3, 20). The thymi of *Pggt1b*^*fl*/*fl*^ *dLckCre* mice exhibited an increased number of CD4 and CD8 SP thymocytes compared to that in *Pggt1b*^*fl*/*fl*^ littermate controls (Figures 1A,C) despite the total number of thymocytes being largely unaffected (Figure 1B). Extra subpopulations of CD69^low^ CD62L^high^ amongst both CD4 and CD8 SP thymocytes emerged in the thymi of the *Pggt1b*^*fl*/*fl*^ *dLckCre* mice (Figures 1A,D,E) that were not present in that of the wild-type littermate controls. This phenotype resembles that in *S1pr1*-deficient mice (3, 20), suggesting that protein geranylgeranylation regulates S1PR1 expression or signaling. An analysis of the S1PR1 expression on thymocytes, particularly the CD69^low^ CD62L^high^ subpopulation from the thymi of the *Pggt1b*^*fl*/*fl*^ *dLckCre* mice, showed that cell surface S1PR1 (Figure 1A) and integrin α4β7 (data not shown) expression was normal, suggesting that Pggt1b deficiency likely results in defective S1PR1 signaling.

### Protein Geranylgeranylation Is Required for T Lymphocyte Homing to Secondary Lymphoid Organs

**Figure 2:**
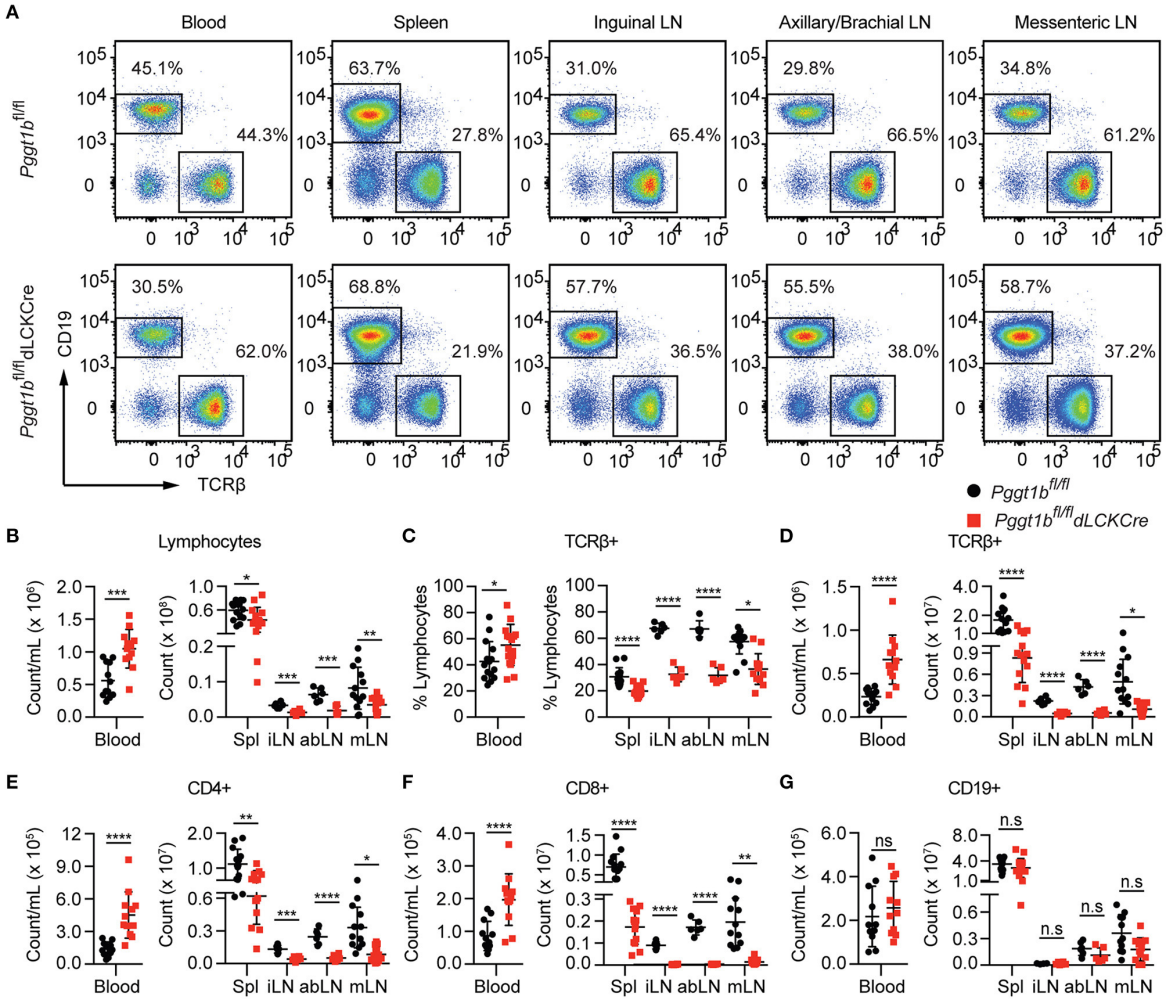
T-Lympopenia in secondary lymphoid organs of *Pggt1b*^*fl*/*fl*^ *dLckCre* mice **(A)** Flow cytometry analysis of CD19 and TCRβ positive cells in the blood, spleen, and lymph nodes; **(B–G)** Total cell number of lymphocytes **(B)**; Percentage **(C)** and number **(D)** of TCRβ^+^ cells; Total number of CD4^+^ **(E)**, CD8^+^ **(F)**, and CD19^+^ **(G)** cells in blood, spleen, and lymph nodes. Each dot represents a single mouse iLN, abLN, mLN: inguinal, axillary, and brachial, mesenteric lymph nodes, respectively (n.s. statistically not significant; *p < 0.05, **p < 0.01, ***p < 0.001, ****p < 0.0001, unpaired *t*-test).

We next investigated homeostatic T cell migration in the periphery. Total cellularity of T cells in the blood of *Pggt1b*^*fl*/*fl*^ *dLckCre* mice was increased while that in the spleen and lymph nodes was decreased compared to *Pggt1b*^*fl*/*fl*^ littermate controls (Figures 2A,B). The decrease of total T lymphocytes cellularity was more profound in lymph nodes than spleen in *Pggt1b*^*fl*/*fl*^ *dLckCre* mice (Figures 2B–F). Phenotypically, Pggt1b-deficient CD4^+^ T cells displayed normal expression of CD62L (data not shown), integrin α4β7 (Supplementary Figure 1H) and CCR7 (Supplementary Figures 2A,B) compared to wild-type control cells. As expected, the cellularity of CD19^+^ B cells in both blood and peripheral lymphoid organs were not altered in *Pggt1b*^*fl*/*fl*^ *dLckCre* mice compared with *Pggt1b*^*fl*/*fl*^ littermate controls (Figure 2G). Taken together, these observations suggest that protein geranylgeranylation is intrinsically required for homeostatic T lymphocyte homing to SLOs in the periphery.

### Defective *in vitro* Transmigration and *in vivo* Homing of Pggt1b-Deficient CD4^+^ T Cells

**Figure 3:**
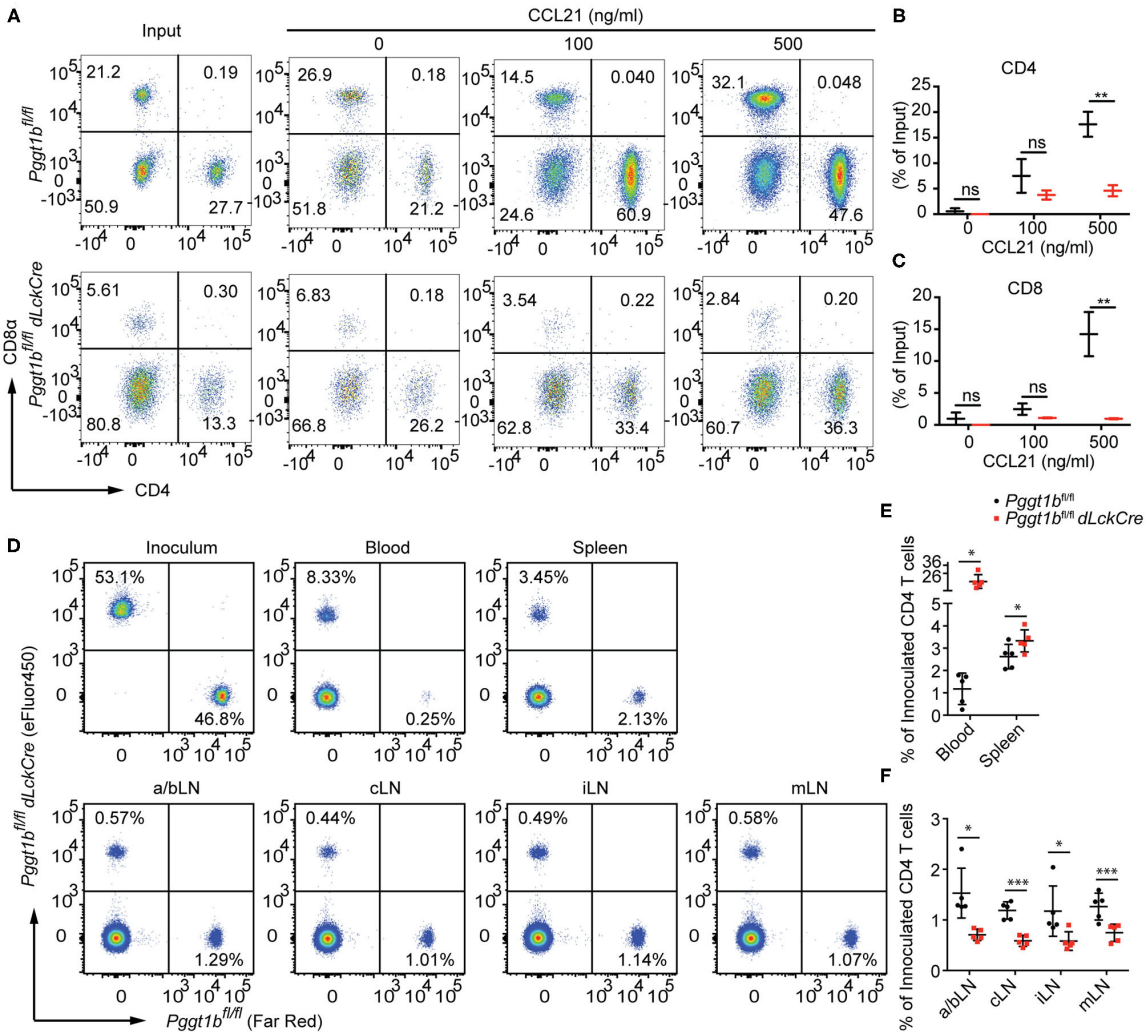
Defective *in vitro* transmigration and *in vivo* homing of Pggt1b-deficient CD4^+^ T cells. **(A)** Flow cytometry analysis of CD4^+^, CD8^+^ T cells in transwell migration assay in response to CCL21; **(B,C)** percentage of input CD4^+^ **(B)** and CD8^+^ **(C)** cells migrated into the low chamber of transwell in response to CCL21; **(D)** Flow cytometry analysis of eFluor450-labelled CD4^+^ naive T cells from *Pggt1b*^*fl*/*fl*^ mice or Far Red-labelled naive CD4^+^ T cells from *Pggt1b*^*fl*/*fl*^ *dLckCre* mice that were mixed at a ratio of 1:1 before injected *i.v*. into recipient mice; **(E,F)** Percentage of CD4^+^ T cells out of the total cells injected in the blood, spleen **(E)** and various lymph nodes **(F)** of the recipient mice. Each dot represents a single mouse. a/b, axillary and brachial; c, cervical; i, inguinal; m, mesenteric lymph nodes (n.s. statistically not significant; *p < 0.05, **p < 0.01, ***p < 0. 001, unpaired *t*-test).

Lymphocyte migration is critically regulated by chemokine-chemokine receptor signaling (2). The impaired T lymphocyte homeostatic homing and thymocyte egress as well as the normal surface expression of S1PR1 on mature thymocytes in *Pggt1b*^*fl*/*fl*^ *dLckCre* mice (Figure 1A) suggest that protein geranylgeranylation is likely required for chemokine-induced migration. To test this, we conducted an *in vitro* transmigration assay. The C-C motif chemokine ligand 21 (CCL21) is an instrumental chemokine essential for T lymphocyte homing to SLOs (2, 21). CD4^+^ and CD8^+^ T cells from the spleen of *Pggt1b*^*fl*/*fl*^ *dLckCre* mice displayed substantially diminished transmigration capacity in response to CCL21 compared to that from wild-type littermate controls (Figures 3A–C). To further assess the capacity of Pggt1b-deficient CD4^+^ T cells in homing to SLOs, a roughly 1:1 mix of differentially labeled purified wild-type and Pggt1b-deficient CD4^+^ naive T cells was administered *i.v*. into recipient C57BL/6 mice. Consistent with the observation in the steady state, the Pggt1b-deficient CD4^+^ naive T cells were retained in the blood stream and their capacity to homing to SLOs, more specifically peripheral lymph nodes, was impaired in the recipient mice (Figures 3D–F). The inability of Pggt1b-deficient T cells in response to CCL21 is not due to decreased expression of CCR7 (Supplementary Figures 2A,B) or impaired survival of these cells *in vitro* (Supplementary Figures 2C,D). Together, those data indicate that Pggt1b-deficient T cells have an intrinsic transmigration defect in response to chemokine CCL21.

### Protein Geranylgeranylation Is Required for Heterotrimeric Small GTPases-Mediated Chemokine Receptor-Proximal Signaling

**Figure 4:**
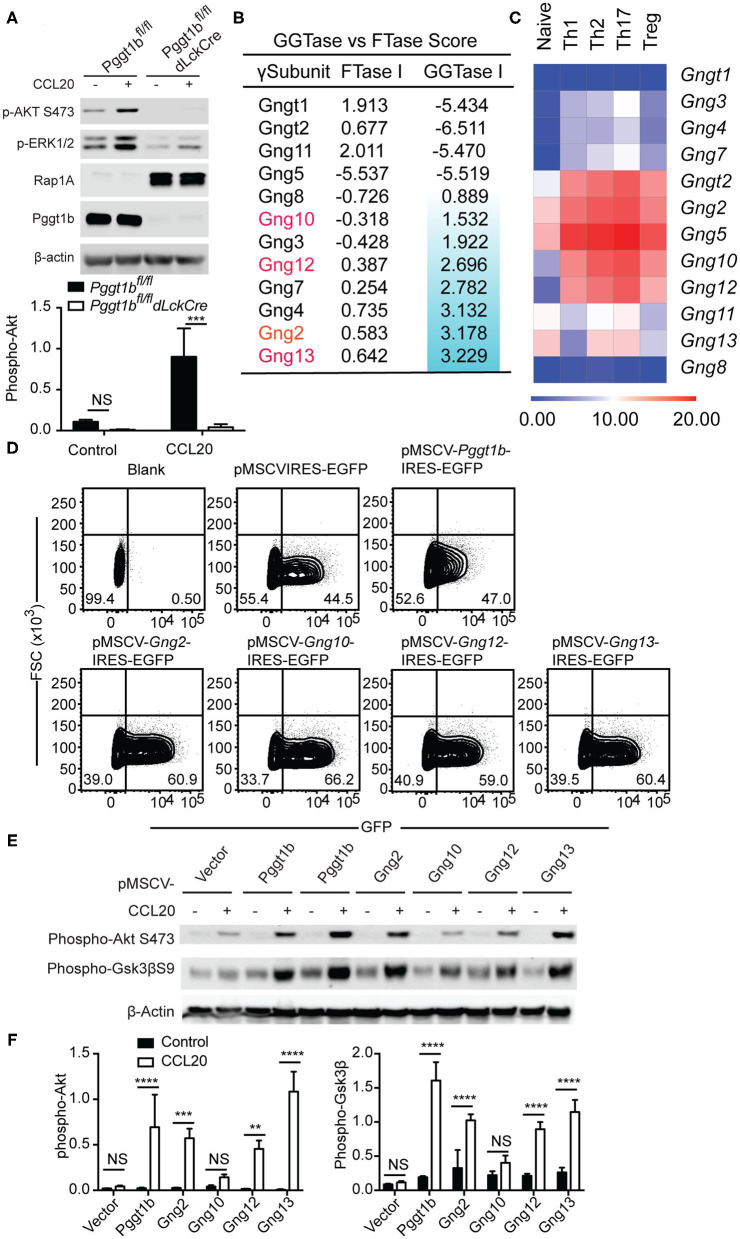
Protein geranylgeranylation is required for heterotrimeric small G-protein mediated GPCR-proximal signaling. **(A)** Western blot analysis of CCL20-induced phosphorylation of Akt and Erk in Th17 cells and density of phosphor-Akt calculated using ImageJ; **(B)** FTase I and GGTase I scores of the 12 γ-subunits of the small heterotrimeric GTPases calculated using an algorithm described in the text; **(C)** Fold change of the expression of the genes encoding the 12 γ-subunits of heterotrimeric small GTPase in naive and effector CD4^+^ T cells analyzed by qRT-PCR; **(D)** Flow cytometry analysis of EGFP expression in Th17 cells infected with retrovirus carrying cDNAs encoding mutant γ-subunits capable of being farnesylated; **(E)** Western blot analysis of phosphor-Akt and phosphor-Gsk3βS9 in Th17 cells described in **(D)**; **(F)** Density of phosphor-Akt and phosphor-Gsk3β in **(E)** calculated using Image J (Results are representatives of three biologically independent experiments, n.s. statistically not significant; **p < 0.01, ***p < 0. 001, ****p < 0. 0001unpaired *t*-test).

The C-C motif chemokine ligand 20 (CCL20) is essential for the migration of Th17 cells into target tissues such as the central nervous system in the context of neuroinflammation (22). Similar to naive T cells, Pggt1b-deficient Th17 cells failed to transmigrate in response to CCL20 despite of CCR6 expression (Supplementary Figures 3A,B). To investigate the underlying molecular mechanism by which protein geranylgeranylation regulates chemokine receptor signaling, we stimulated *in vitro* differentiated Th17 cells with CCL20. We chose Th17 cells to further study chemokine receptor signaling since the proliferative Th17 cells are easier than naive T cells for retroviral infection for genetic complementation approaches. CCL20 stimulation induced robust phosphorylation of Akt, as well as Erk that was impaired in Pggt1b-deficient Th17 cells (Figure 4A). Both Akt and Erk phosphorylation are early signaling events proximal to chemokine receptors necessary for the initiation of cellular signaling cascades required for migration (6). The impaired CCL20-induced Akt and Erk activation in Pggt1b-deficient cells demonstrates that protein geranylgeranylation is indeed required for chemokine receptor proximal signaling.

Chemokine receptors such as CCR6, CCR7, and S1PR1 are G-protein coupled receptors (GPCRs) that use small heterotrimeric GTPases to relay signals initiated at the plasma membrane (23). Among the three heterotrimeric (α*βγ*) subunits of small G-proteins, prenylation [i.e., geranylgeranylation and farnesylation (8)] of the γ-subunits is required for the plasma membrane localization and functional activity of the small GTPases complex (24). We reasoned that the absence of protein geranylgeranylation would abrogate the plasma membrane localization of γ-subunits of small GTPases and therefore impair chemokine receptor-proximal signaling.

There are 12 γ-subunits encoded by 12 distinct genes in the murine genome. Precisely, which γ-subunits mediates T cell migration remains largely unresolved. We extracted the microarray expression data of the genes encoding the 12 γ-subunits in murine αβ T cells from the Immgen consortium (25). The array data suggested that only *Gng t2, 2, 4, 5, 10, 12*, and *13* were expressed in αβ CD4^+^ T cells (Supplementary Figure 3C) which we then verified by quantitative RT-PCR. We confirmed that *Gng t2, 2, 5, 10, 12, 13* were highly expressed in naive and effector CD4^+^ T cell subsets but observed that *Gng4* had a lower expression level than predicted by the array data (Figure 4C). We next calculated the prenylation scores of the 12 γ-subunits using a web-based algorithm (26). Of the 6 γ-subunits highly expressed in T cells, Gng13, 2, 12, and 10 have positive geranylgeranylation scores in a descending order (Figure 4B), while Gngt2 and Gng5 have negative geranylgeranylation scores (Figure 4B). We reasoned that the 4 γ-subunits, Gng2, Gng10, Gng12, Gng13, that are highly expressed in αβ T cells with positive geranylgeranylation scores are likely the ones that mediate chemokine receptor signaling in T lymphocytes. To test this hypothesis, we swapped the CAAX (8) motifs in cDNAs encoding Gng2, Gng10, Gng12, Gng13 with sequences encoding a CVSL motif that has been previously shown capable of being farnesylated and restoring the function of Rho GTPases in Pggt1b-deficient macrophages (10, 11, 21, 27). We designated these mutant forms of γ-subunit as “farnesylable.” The cDNAs encoding the “farnesylable” forms of the 4 γ-subunits were cloned into a bi-cistronic retroviral vector expressing an IRES-driven eGFP reporter and transduced into Pggt1b-deficient Th17 cells. Successful transduction was determined by eGFP expression (Figure 4D). Retrovirus-mediated ectopic expression of Pggt1b, or the “farnesylable” Gng2, Gng12, Gng13 γ-subunits resulted in the rescuing of Akt and Gsk3β phosphorylation while expression of Gng10 failed to do so (Figures 4E,F). However, farnesylable Gng13 reconstituted Pggt1b-deficient T cells failed to migrate in response to CCL20 *in vitro*, suggesting that protein geranylgeranylation controls additional signaling components in addition to small trimeric GTPases (Supplementary Figure 3D). Since heterotrimeric GTPases directly link GPCR triggering to receptor-proximal signaling, these data suggest that geranylgeranylation likely controls chemokine receptor-proximal signaling through modifying the Gng2, Gng12, and Gng13 γ-subunits of the heterotrimeric small GTPases.

### *Pggt1b^*fl*/*fl*^ dLckCre* Mice Are Resistant to EAE Induction

We next tested how protein geranylgeranylation controls effector T cell function. In a mouse model of experimental autoimmune encephalomyelitis (EAE), encephalitogenic T cells primed in the periphery migrate to the central nervous system to orchestrate pathological changes (28) by recruiting other inflammatory cells such as monocyte-derived dendritic cells (MoDCs) (29, 30). EAE resembles many of the immuno-pathological features of human multiple sclerosis and provides an excellent model for testing effector T cell migration and function. The trafficking of pathogenic immune cells during EAE is subjected to the regulation by chemokine and chemokine receptor signaling (22, 31). The S1P1 signaling antagonist Fingolimod has been approved for treating human MS as it sequesters effector T cells within the lymph nodes (32–34).

**Figure 5:**
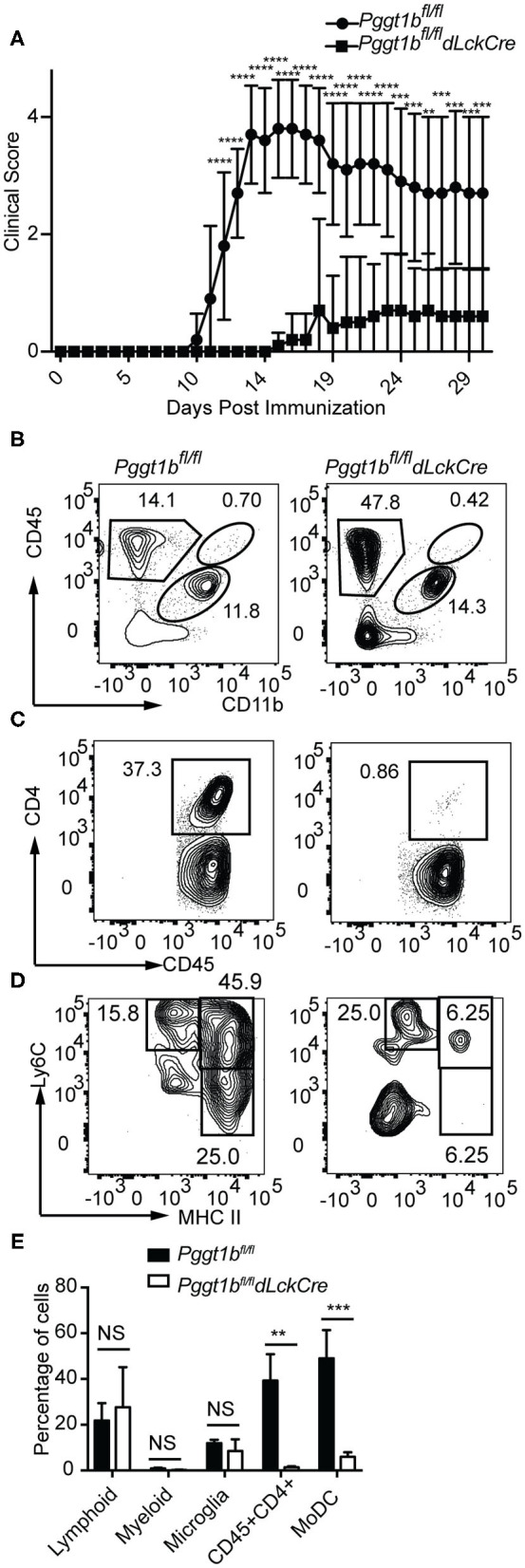
*Pggt1b*^*fl*/*fl*^ *dLckCre* mice are resistant to EAE induction. **(A)** Clinical scores of mice immunized with MOG35-55 peptide emulsified in complete Freud's adjuvant; **(B–D)** Flow cytometry analysis of spinal cords leukocytes isolated from mice on day 19 after immunization and stained with antibodies against CD45, CD11b, CD4, Ly6C, Ly6G, CD44, CD64, and MHC II and gated according to a strategy described in the text to distinguish myeloid, lymphoid, microglia, CD4^+^ T cells, and monocyte-derived dendritic cells (MoDCs); **(E)** Percentage of lymphoid, myeloid, microglia, CD4^+^ T cells, and MoDCs in the spinal cord (Results are from two independent biological experiments with a total of 20 mice (10 male, 10 female)) [NS, not significant, **p < 0.01, ***p < 0.001, ****p < 0.0001, way anova **(A)**, unpaired *t*-test **(E)**].

EAE was induced by immunization of mice with myelin oligodendrocyte glycoprotein (MOG) peptide 35–55 emulsified in complete Freud's adjuvant. Leukocytes were isolated from spinal cords, stained and gated according to a strategy described by Caravagna et al. (17). While *Pggt1b*^*fl*/*fl*^ mice developed EAE as defined by clinical scores, *Pggt1b*^*fl*/*fl*^ *dLckCre* mice were resistant to EAE induction (Figure 5A). Consistent with the clinical scores, at the onset (day 14, Supplementary Figure 4) or the peak of the disease (day 19, Figures 5B–E), CD4^+^ T cells as well as the pathogenic Ly6C^high^ MHC II^high^ MoDCs (29, 30) were recruited into the CNS in *Pggt1b*^*fl*/*fl*^ wild-type littermate controls, but not in *Pggt1b*^*fl*/*fl*^ *dLckCre* mice.

**Figure 6:**
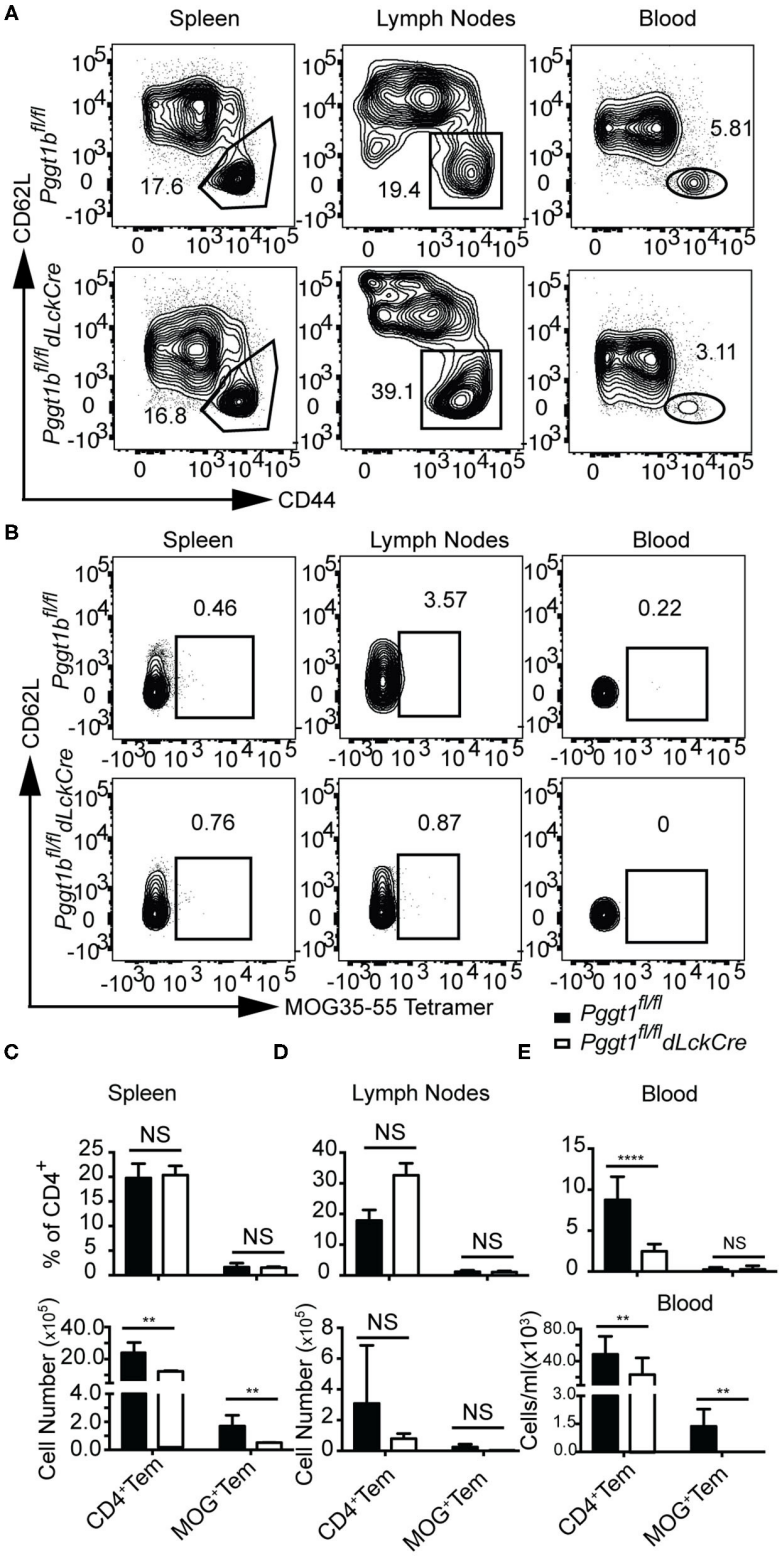
Defective effector T cell egress from secondary lymphoid organs after primary immunization in *Pggt1b*^*fl*/*fl*^*dLckCre* mice. **(A)** Flow cytometry analysis of effector (CD44^high^, CD62L ^low^) CD4^+^ T cells in the spleen, draining lymph nodes and blood 7 days after immunization; **(B)** Flow cytometry analysis of MOG-specific CD4^+^ effector T cells in spleen, lymph nodes and blood 7 days after immunization; absolute number of CD4^+^ effector cells and MOG-specific CD4^+^ effector T cells in spleen **(C)**, lymph nodes **(D)**, and blood **(E)**. Results are representative of two biologically independent experiments with a total of 16 (8 male, 8 female) mice (NS, not significant, **p < 0.01, ***p < 0.001, ****p < 0.0001 unpaired *t*-test).

In EAE, primed encephalitogenic T cells emigrate from SLOs and travel through the circulation to the CNS to orchestrate autoimmunity (35). Emigration of effector T cells from SLOs after priming also depends on chemokine receptor signaling (2, 36). Given that there were very few CD4^+^ T cells found in the CNS of *Pggt1b*^*fl*/*fl*^ *dLckCre* mice during EAE induction, we reasoned that either the egress from SLOs or their entry into the CNS of effector T cells was impaired in the mutant mice. In steady state, Pggt1b-deficient naive CD62L^high^ CD44^lo^ CD4^+^ cells accumulated in the circulation and were impaired in homing to SLOs (Supplementary Figures 6A,B). Whereas, the absolute number of CD4^+^ CD62L^lo^ CD44^high^ memory/effector (Tem) cells in the blood in *Pggt1b*^*fl*/*fl*^ *dLckCre* mice was similar to that in wild-type control mice, the number of Tem cells in SLOs seemed to be reduced in *Pggt1b*^*fl*/*fl*^ *dLckCre* mice than that in wild-type control mice (Supplementary Figures 6C,D). MOG immunization increased the frequency of Tem CD4^+^ T cells in lymph nodes but not spleen in both mouse strains in comparison to steady-state mice (Supplementary Figures 6C,D and Figures 6C,D), indicating that the priming was not substantially affected in the lymph nodes of *Pggt1b*^*fl*/*fl*^ *dLckCre* mice. However, the frequency and number of Tem cells in the blood of *Pggt1b*^*fl*/*fl*^ *dLckCre* mice were significantly lower than those in *Pggt1b*^*fl*/*fl*^ littermate controls (Figures 6A,E) 7 days after immunization. More importantly, MOG35-55-specific CD4^+^ Tem cells (encephalitogenic T cells) were barely detectable in the blood of *Pggt1b*^*fl*/*fl*^ *dLckCre* mice after immunization compared with that in wild-type littermate controls (Figures 6B,E). Those observations suggest that protein geranylgeranylation is required for effector T cell emigration from SLOs in a primary adaptive immune response.

We further determined whether *in vitro* differentiated Pggt1b-deificient pathogenic Th17 cells could cause EAE. Recipient mice adoptively transferred with Pggt1b-deficient 2D2 (MOG-specific TCR)-transgenic (15) Th17 cells failed to develop EAE, whereas mice received wild-type 2D2-transgenic Th17 cells developed clinical diseases accompanied by severe body weight loss (Supplementary Figure 5).

### Pggt1b-Deficient CD4^+^ Naive T Cells Preferentially Differentiated Into Foxp3^+^ T Regulatory Cells at the Cost of Th17 Cells *in vitro*

**Figure 7:**
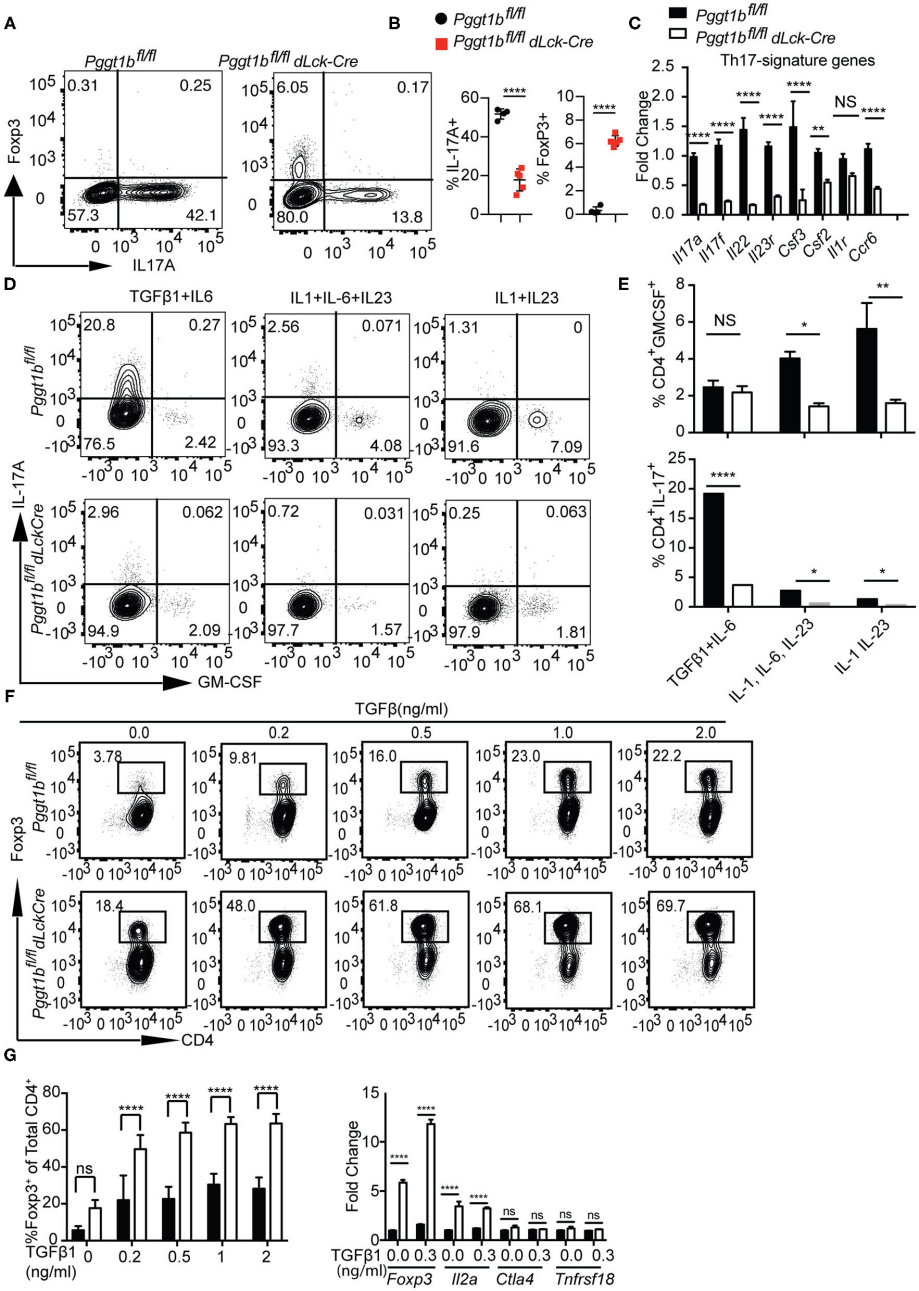
Pggt1b-deficient naive T cells are predisposed to differentiate into T regulatory cells *in vitro*. **(A)** Flow cytometry analysis of the expression of Foxp3 and IL-17A in cells cultured in the presence of plate-bound anti-CD3 and anti-CD28 antibodies and TGFβ1 and IL-6 for 72 h; **(B)** Percentage of IL-17A+ or Foxp3+ CD4+ cells in the culture described in **(A)**; **(C)** Fold change of Th17 signature cytokines in cells described in **(A)** analyzed by qRT-PCR; **(D)** Flow cytometry analysis of GM-CSF^+^ and IL-17^+^ CD4^+^ T cells in cells cultured in the presence of TGFβ1 and IL-6, IL-1, IL-6, and IL-23 or IL-1 IL-23; **(E)** Percentage of GM-CSF^+^ and IL-17A^+^ CD4^+^ T cells in cultures described in **(D)**; **(F)** Flow cytometry analysis of naive T cells 72 h after cultured in the presence of plate-bound anti-CD3 and anti-CD28 antibody and different concentrations of TGFβ1; **(G)** percentage of Foxp3+ CD4+ T cells in the culture described in **(F)** and qRT-PCR analysis of the expression T regulatory cell genes in cell cultures described in **(F)**. Results are representative of three independent biological experiments [n.s. statistically not significant; *p < 0.05, **p < 0.01, ***p < 0.001, ****p < 0.0001, two-way anova **(C)**, unpaired *t*-test **(G)**].

Th17 cells play important roles in the host resistance to extracellular pathogens and in the pathogenesis of autoimmune diseases (37). Th17 cell early differentiation is driven by transforming growth factor-β (TGFβ1) and IL-6 (38–40). When cultured in the presence of TGFβ1 and IL-6, the differentiation of Th17 cells from Pggt1b-deficient naive CD4^+^ T cells was impaired compared to that from wild-type controls (Figure 7A). More strikingly, this defect was concomitant with the emergence of iTregs in the culture (Figures 7A,B). In addition to IL-17A, mRNA expression of other Th17 signature cytokines were also significantly lower in Pggt1b-deficient Th17 cell cultures than that in wild-type controls (Figure 7C). Th17 cells need exposure to IL-23 to gain pathogenicity in an EAE model (41–43). Together with the pro-inflammatory cytokine IL-1, IL-23 promotes the differentiation of Th17 cells into GM-CSF-producing effector cells (44) that recruit pathogenic MoDCs into the CNS to cause EAE (45, 46). When cultured in the presence of IL-1 and IL-23 or IL-1, IL-6, and IL-23, Pggt1b-deficient CD4^+^ naive T cells were also defective in the differentiation into GM-CSF-producing effector cells *in vitro* (Figures 7D,E).

The increased Foxp3^+^ iTreg cells found in Th17 cultures prompted us to investigate whether *in vitro* differentiation of iTregs was also affected by Pggt1b deficiency. Pggt1b-deficient naive CD4^+^ T cells exhibited significantly enhanced differentiation into Foxp3^+^ iTregs in the presence of low concentrations of TGFβ1 with increased expression of *Foxp3* as well as *IL2a*, but not *Ctla4* and *Tnfrsf18* compared with wild-type control cells (Figures 7F,G). In accordance with these findings, IL-6-stimulated phosphorylation of Stat3, a transcription factor essential for Th17 cell differentiation (47, 48), was impaired in Pggt1b-deficient CD4^+^ naive T cells while TGFβ1-induced phosphorylation of Smad2 and Smad3, transcription factors pivotal to iTreg differentiation (49), was enhanced (Supplementary Figures 7A–C). We conclude that protein geranylgeranylation is a critical determinant in the balance of lineage commitment for naive CD4^+^ T cells to Th17 or iTreg cells *in vitro*. Nevertheless, the development of nTregs in the thymus of Pggt1bfl/fldLckCre mice was largely unaltered (Supplementary Figure 7D).

## Discussion

Protein geranylgeranylation is a post-translational lipid modification that regulates diverse processes ranging from cell cycle progression to innate immune response. Using a mouse strain with T cell lineage-specific ablation of the β-subunit of GGTase-I, we demonstrate that protein geranylgeranylation controls T cell migration by regulating chemokine-receptor-proximal signaling. Protein geranylgeranylation also promotes the development of inflammatory Th17 cells while inhibiting naive T cells differentiation into Foxp3^+^ iTreg cells *in vitro*.

Migration between different compartments of the thymus is crucially required for thymocyte development (50). Two previous studies (12, 13) reported severe lymphopenia in the periphery in *Pggt1b*^*fl*/*fl*^*CD4Cre* mice. The CD4-promoter/enhancer/silencer-driven cre induces the deletion of loxp sites-flanked genes with >90% efficiency in DP thymocytes (51). DP thymocytes need to migrate from the cortex of thymus to the medulla while continuing maturation into CD4 or CD8 SP thymocytes. This migration is driven by chemokine receptor signaling (52). Given the pivotal role of Pggt1b in chemokine receptor signaling, abrogation of Pggt1b in DP thymocytes would presumably impede the migration of those cells from cortex to medulla and likely impair their maturation. We have established a mouse strain in which cre-mediated deletion of *Pggt1b* occurs at late stage of thymocyte development by utilizing *dLckCre* transgene (14). Our data showed that cre-mediated deletion of *Pggt1b* occurs only in a very small fraction of DP thymocytes. Though we observed increased numbers of mature thymocytes in the thymus, a significant number of mature naive T cells migrated into periphery, presumably before the completion of cre-mediated deletion of *Pggt1b* as well as a thorough degradation of existing geranylgeranylated proteins. Instead, we observed accumulation of naive T cells in the blood and a defect of Pggt1b-defcient mature T cells in homing to SLOs. Thus, our animal model enabled us to study how protein geranylgeranylation regulates the migration and function of mature T cells in the periphery without severely impeding early thymocyte development.

Lopez-Posadas et al. reported that the majority of Pggt1b-deficient CD4 T cells in *Pggt1b*^*fl*/*fl*^*CD4Cre* mice displayed an activated phenotype with up-regulation of α4β7 and CD44 (13) as well as localization to colon with increased expression of inflammatory cytokines that caused colitis (12). However, those phenotypes were not recapitulated in *Pggt1b*^*fl*/*fl*^*dLckCre* mice. Instead, naive Pggt1b-deficient CD4^+^ T cells from *Pggt1b*^*fl*/*fl*^*dLckCre* mice preferentially differentiated into iTregs at the cost of Th17 cell differentiation *in vitro*. This discrepancy is likely because deletion of Pggt1b at an early stage of thymocyte development in *Pggt1b*^*fl*/*fl*^*CD4Cre* mice may have altered the TCR repertoire or property that these CD4^+^ T cells become colitogenic. While not explored by López-Posadas et al. (12) and Du et al. (13) in *Pggt1b* ^*fl*/*fl*^*CD4Cre* mice, we observed normal ratio of nTregs in the thymus (Supplementary Figure 7D) and periphery (data not shown) in *Pggt1b* ^*fl*/*fl*^*dLckCre* mice. The unaffected nTreg development as well as the propensity of naive T cells to differentiate into iTregs likely helped maintain the immune homeostasis in the colon in *Pggt1b* ^*fl*/*fl*^*dLckCre* mice.

Chemokine and chemokine receptor signaling guides the migration of lymphocytes which is essential for immune surveillance and successful adaptive immune responses. However, signaling pathways downstream of chemokine receptors remain poorly understood. It is known that protein geranylgeranylation is essential for the function of Rho family small GTPases such as Rac, Rho, and Cdc42 (8, 53), critical players in signaling distal to chemokine receptors. Deficiency of RhoA specifically in T cells also resulted in reduced T cell function (54). It has also been reported that protein geranylgeranylation promotes Cdc42 and Pak signaling and Tiam1 expression in T cells (13, 37). However, RhoA, Pak and Tiam1-dependent signaling events are distal to chemokine-receptor and secondary to the receptor-proximal signaling. Data presented here demonstrate that, in addition to the above mentioned distal signaling events, protein geranylgeranylation also controls the early signaling events immediately downstream of chemokine receptors through modification of the γ-subunits of heterotrimeric small GTPases.

Prenylation of the γ-subunit is essential for the plasma membrane-localization and signal-relaying function of heterotrimeric small GTPases (24). Among the 12 γ-subunits, eight of them are prone to protein geranylgeranylation; three of them are likely to be farnesylated and one can be neither farnesylated nor geranylgeranylated based on the web-based prenylation scoring algorithm (26). It has been reported that abrogation of farnesylation in T cells does not affect thymocyte egress (13). However, it is highly likely that farnesylation may play a role in regulating the migration of immune cells other than T lymphocytes. It is also of note that when farnesylation is inhibited, some farnesylation-prone substrate can be modified by protein geranylgeranylation and become fully functional (55) and this may impede the study of intrinsic role of protein farnesylation in cell migration or other physiological processes. Nevertheless, our results support a critical role of protein geranylgeranylation in regulating mature T lymphocyte migration.

The high endothelial venules (HEV), a highly specialized type of post capillary endothelium within the paracortical regions, play important role in lymphocyte entry into lymph nodes (56). Transmigration of T cells through HEV to enter lymph nodes is a regulated process that is dependent on chemokine-chemokine receptor signaling. In contrast, there is no HEV in spleen. T cells enter the red pulp of the spleen where the central arterioles are open through an unregulated, passive process. Our results showed that more Pggt1b-deficient T cells homed to spleen than the wild-type control cells in the *in vivo* homing assay. This is likely because Pggt1b-deficient T cells can enter the spleen red pulp as efficient as wild-type cells. The increased number of Pggt1b-deficient T cells in the circulation might have also enhanced the effect.

Given the fact that Pggt1b-deficient naive T cells homing to SLOs is impaired, the efficiency of T lymphocyte priming after immunization would also be adversely affected in *Pggt1b*^*fl*/*fl*^ *dLckCre* mice. This is one of the limitations of using the current animal model to study immunization efficiency. However, the post-immunization frequency of Tem cells in SLOs were similar between *Pggt1b*^*fl*/*fl*^*dLckCre* mice and the wild-type littermate controls (Figures 6C,D) although the absolute numbers were significantly lower in *Pggt1b*^*fl*/*fl*^*dLckCre* mice. This could be the result of combined effects of the impaired entry of naive T cells as well as the deficient egress of effector T cells in a dynamic environment. In addition, protein geranylgeranylation is also likely to impact the priming efficacy by affecting the interaction between naive T cells and antigen presenting cells in SLOs since Rho family small GTPases, important regulators of immunological synapse formation (16), are prototypes of GGTase-I substrates. However, the result that *in vitro* differentiated 2D2-transgenic Pggt1b-deficient Th17 cells, which by-passed the *in vivo* priming process, failed to induce EAE in recipient mice, indicates that protein geranylgeranylation controls immune responses beyond priming in SLOs. Given the intrinsic defect of naive Pggt1b-deficint T cells in Th17 cell differentiation and transmigration, α47 the observed phenotype of decreased EAE score in *Pggt1b*^*fl*/*fl*^*dLckCre* mice is likely due to the combined effects of impaired migration and compromised effector function of Th17 cells. Future experiments are warranted to disentangle those two impacts caused by Pggt1b-deficiency.

T cell egress from SLOs also depends on S1PR1 signaling (36). S1PR1 is a GPCR that also depends on trimeric small GTPases for signal transduction which requires protein geranylgeranylation. Since integrin activation is downstream of chemokine receptor signaling in the inside-out signaling cascade, a defective chemokine receptor signaling would inevitably lead to compromised integrin activation. The S1P1 signaling antagonist Fingolimod has been approved for treating human MS as it sequesters effector T cells within the lymph nodes (32–34, 57). Therefore, the EAE model with *Pggt1b* ^*fl*/*fl*^*dLckCre* mice resembles those of mice treated with S1P1 functional antagonist. The observation that there was substantial number of effector T cells in SLOs after immunization, the significantly decreased number of effector T cells in the blood, and the near absence of MOG-specific effector T cells in the circulation despite of the presence of these cells in SLOs in *Pggt1b*^*fl*/*fl*^ *dLckCre* mice, combined with the fact that effector T cell emigration from SLOs depends on S1PR1 signaling, strongly suggested that protein geranylgeranylation is likely required for the emigration of effector T cells into the circulation after primary immunization.

Protein geranylgeranylation utilizes geranylgeranyl pyrophosphate (GGPP) as a substrate. GGPP is an intermediate product of the cholesterol-synthesizing mevalonate pathway. Therefore, our finding revealed a metabolic control of lymphocyte migration and effector function by a fundamental metabolic pathway. Elucidation of the molecular mechanisms by which lipid metabolism controls immune cell function may provide new opportunity in managing immune-mediated diseases or for manipulations to enhance immune cell function in treating cancer.

## Data Availability Statement

The raw data supporting the conclusions of this article will be made available by the authors, without undue reservation.

## Ethics Statement

The animal study was reviewed and approved by IACUC, Duke University.

## Author Contributions

DW conceived the research. DW, GS, JG, and GEH contributed to experimental design and evaluation of results. GS, JG, EP, QD, JJZ, GS, and CW conducted experiments. JZ and H-IH helped with flow panel set up and flow cytometry data analysis. DW drafted the manuscript. GS and EP worked on editing, revising, and finalizing the manuscript. All authors contributed to the article and approved the submitted version.

## Conflict of Interest

The authors declare that the research was conducted in the absence of any commercial or financial relationships that could be construed as a potential conflict of interest.

## Supplementary Material

Supplementary Figure 1 Establishment of the *Pggt1b*^*fl*/*fl*^ *dLckCre* mouse strain. **(A)** Flow cytometry of total thymocytes from *Pggt1b*^*fl*/*fl*^ *tdTomato*^*Stopfloxed*^*dLckCre* mice; **(B,C)** Expression of tdTomato in different subpopulations of thymocytes as described in **(A)**; **(D)** quantitative RT-PCR analysis of Pggt1b expression in FACS-sorted subpopulations of thymocytes as described in **(A)**; **(E)** Immunoblot of total thymocyte lysate; **(F,G)** quantitative RT-PCR analysis **(F)** and immunoblot **(G)** of Pggt1b in CD4^+^ Naive T cells from spleen; **(H)** Flow cytometry analysis of the expression of integrin α4β7 on CD4^+^ naive T cells (data are representative of results of at least two independent biological experiments, n.s. statistically not significant; ****p* < 0.001, *****p* < 0.0001 unpaired *t*-test).

Supplementary Figure 2 CCR7 expression and *in vitro* survival of Pggt1b-deficient naive T cells. **(A)** Flow cytometry analysis of CCR7 expression on naive CD4^+^ T cells; **(B)** Mean fluorescence intensity of CCR7 described in **(A)**; **(C)** percentage and **(D)** number of viable naive CD4^+^ T cells cultured *in vitro* in the absence or presence of mIL-7 (Results are representatives of at least two biologically independent experiments. n.s. statistically not significant; **p* < 0.05, ***p* < 0.01, ****p* < 0.001, *****p* < 0.0001, unpaired *t*-test).

Supplementary Figure 3 Transmigration and CCR6 expression of Th17 cells and the expression of genes encoding the γ-subunits of heterotrimeric small GTPases in αβ T cells. **(A)** Percentage of input of Th17 cells transmigrated into the low chamber in response to CCL20 in transmigration assay; **(B)** Overlay of histogram of flow cytometry analysis of the expression of CCR6 on *in vitro* derived Th17 cells; **(C)** Array expression data were extracted from the Immgen consortium website and converted into logarithmic fold of changes and heatmap were generated using Morpheus web-based tools created by Broad Institute (https://software.broadinstitute.org/morpheus/); **(D)** percentage of input of GFP-positive, vector or Gng13-transduced Pggt1b-deficient Th17 cells transmigrated into the lower chamber in response to 500 ng/ml CCL20.

Supplementary Figure 4 Defective CD4 and monocyte-derived DC infiltration into the CNS of *Pggt1b*^*fl*/*fl*^ *dLckCre* mice on day 14 after immunization. Mice were immunized as described in Figure 5, **(A–C)** Flow cytometry analysis of spinal cords leukocytes harvest on day 19 after immunization that were stained with antibodies against CD45, CD11b, CD4, Ly6C, Ly6G, CD44, CD64, and MHC II and gated according to a strategy described in the text to distinguish myeloid, lymphoid, microglia, CD4^+^ T cells, and monocyte-derived dendritic cells (MoDCs); **(D)** Percentage of lymphoid, myeloid, microglia, CD4+, and MoDCs in the spinal cord (Results are from two independent biological experiments with a total of 20 mice (10 male, 10 female) (NS, not significant, **p* < 0.05, ***p* < 0.01, unpaired *t*-test).

Supplementary Figure 5 Adoptively transferred 2D2-transgenic Pggt1b-deficient Th17 cells failed to induce EAE in recipient mice. **(A)** Clinical score of mice receiving *in vitro* differentiated inflammatory 2D2-transgenic Th17 cells; **(B)** Body weight change of mice described in **(A)**.

Supplementary Figure 6 Naive and effector/memory CD4+ T cells in the periphery. Percentage and number of naive **(A,B)** and effector/memory **(C,D)** CD4^+^ T cells in blood, spleen, inguinal (iLN), auxiliary and brachial (a/bLN) lymph nodes (Each dot represents an individual mouse, ns, not significant, **p* < 0.05, ***p* < 0.01, ****p* < 0.001, unpaired *t*-test).

Supplementary Figure 7 Impaired IL-6-induced Stat3 phosphorylation and enhanced TGFβ1-induced Smad2 and Smad3 phosphorylation in Pggt1b-deficient naive CD4+ T cells. **(A)** Immuno-blot of naive CD4^+^ T cells stimulated with IL-6 with antibodies indicated in the figure; **(B)** Image J calculation of the phosphor-Stat3 as indicated in **(A)**; **(C)** Western blot of phosphor-Smad2 and Smad3 of CD4^+^ naive T cells after TGFβ11 stimulation; **(D)** Flow cytometry analysis of thymic T regulatory cells.
