# Corrigendum: A Requirement of Protein Geranylgeranylation for Chemokine Receptor Signaling and Th17 Cell Function in an Animal Model of Multiple Sclerosis

**DOI:** 10.3389/fimmu.2021.687135

**Published:** 2021-04-19

**Authors:** Gregory Swan, Jia Geng, Eunchong Park, Quanquan Ding, John Zhou, Ciana Walcott, Junyi J. Zhang, Hsin-I. Huang, Gianna Hammer, Donghai Wang

**Affiliations:** ^1^ Division of Rheumatology and Immunology, Department of Medicine, Duke University School of Medicine, Durham, NC, United States; ^2^ Department of Immunology, Duke University School of Medicine, Durham, NC, United States

**Keywords:** protein geranylgeranylation, adaptive immune response, T cells, autoimmunity, lymphocyte migration

In the original article, there was a mistake in **Figure 2A** as published. Incorrect representative flow cytometry graphs were used owing to an error in preparing the figure. The corrected **Figure 2** appears below.

**Figure 2:**
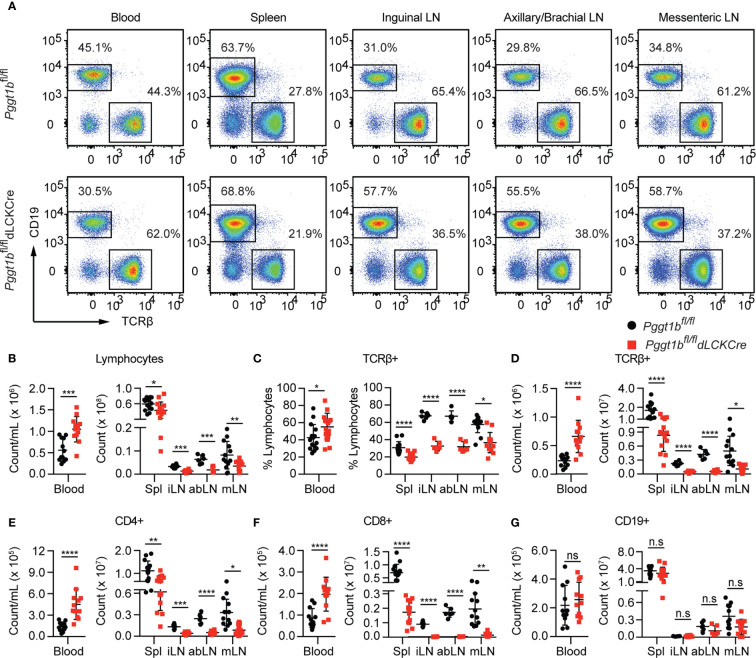
T-Lympopenia in secondary lymphoid organs of *Pggt1b^fl/fl^ dLckCre* mice **(A)** Flow cytometry analysis of CD19 and TCRβ positive cells in the blood, spleen, and lymph nodes; **(B–G)** Total cell number of lymphocytes **(B)**; Percentage **(C)** and number **(D)** of TCRβ^+^ cells; Total number of CD_4_^+^ **(E)**, CD8^+^ **(F)**, and CD_19_^+^ **(G)** cells in blood, spleen, and lymph nodes. Each dot represents a single mouse iLN, abLN, mLN: inguinal, axillary, and brachial, mesenteric lymph nodes, respectively (n.s. statistically not significant; *p < 0.05, **p < 0.01, ***p < 0.001, ****p < 0.0001, unpaired t-test).

The authors apologize for this error and state that this does not change the scientific conclusions of the article in any way. The original article has been updated.

