# Supplementary figures and images for: A Requirement of Protein Geranylgeranylation for Chemokine Receptor Signaling and Th17 Cell Function in an Animal Model of Multiple Sclerosis
Source: Front Immunol. 2021 Mar 22;12:641188. doi: 10.3389/fimmu.2021.641188 (PMC8019753; doi:10.3389/fimmu.2021.641188)

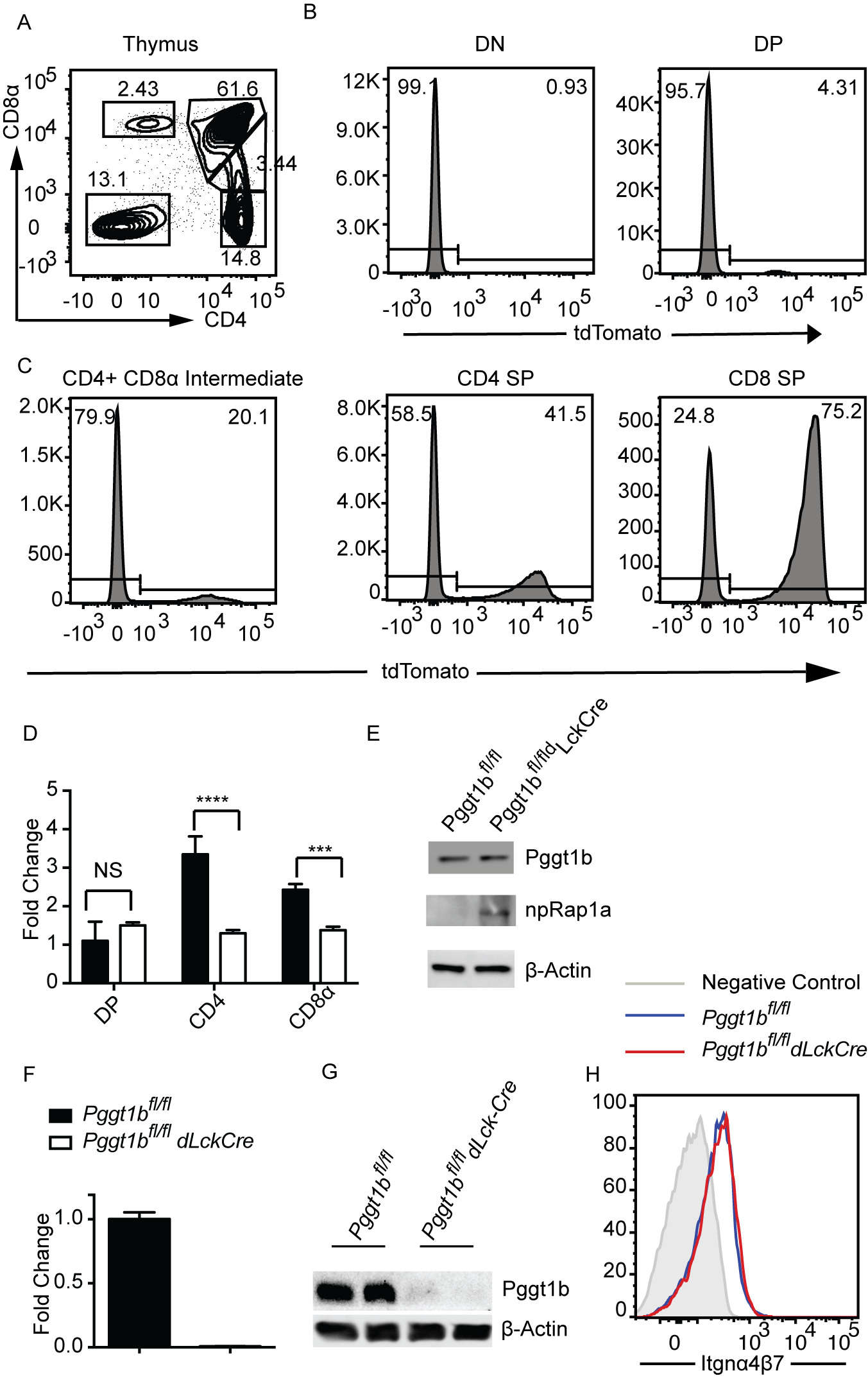

Supplement: Supplementary Figure 1 — Establishment of the Pggt1bfl/fl dLckCre mouse strain. (A) Flow cytometry of total thymocytes from Pggt1bfl/fl tdTomatoStopfloxeddLckCre mice; (B,C) Expression of tdTomato in different subpopulations of thymocytes as described in (A); (D) quantitative RT-PCR analysis of Pggt1b expression in FACS-sorted subpopulations of thymocytes as described in (A); (E) Immunoblot of total thymocyte lysate; (F,G) quantitative RT-PCR analysis (F) and immunoblot (G) of Pggt1b in CD4+ Naive T cells from spleen; (H) Flow cytometry analysis of the expression of integrin α4β7 on CD4+ naive T cells (data are representative of results of at least two independent biological experiments, n.s. statistically not significant; ***p < 0.001, ****p < 0.0001 unpaired t-test). [file Image_1.TIF]

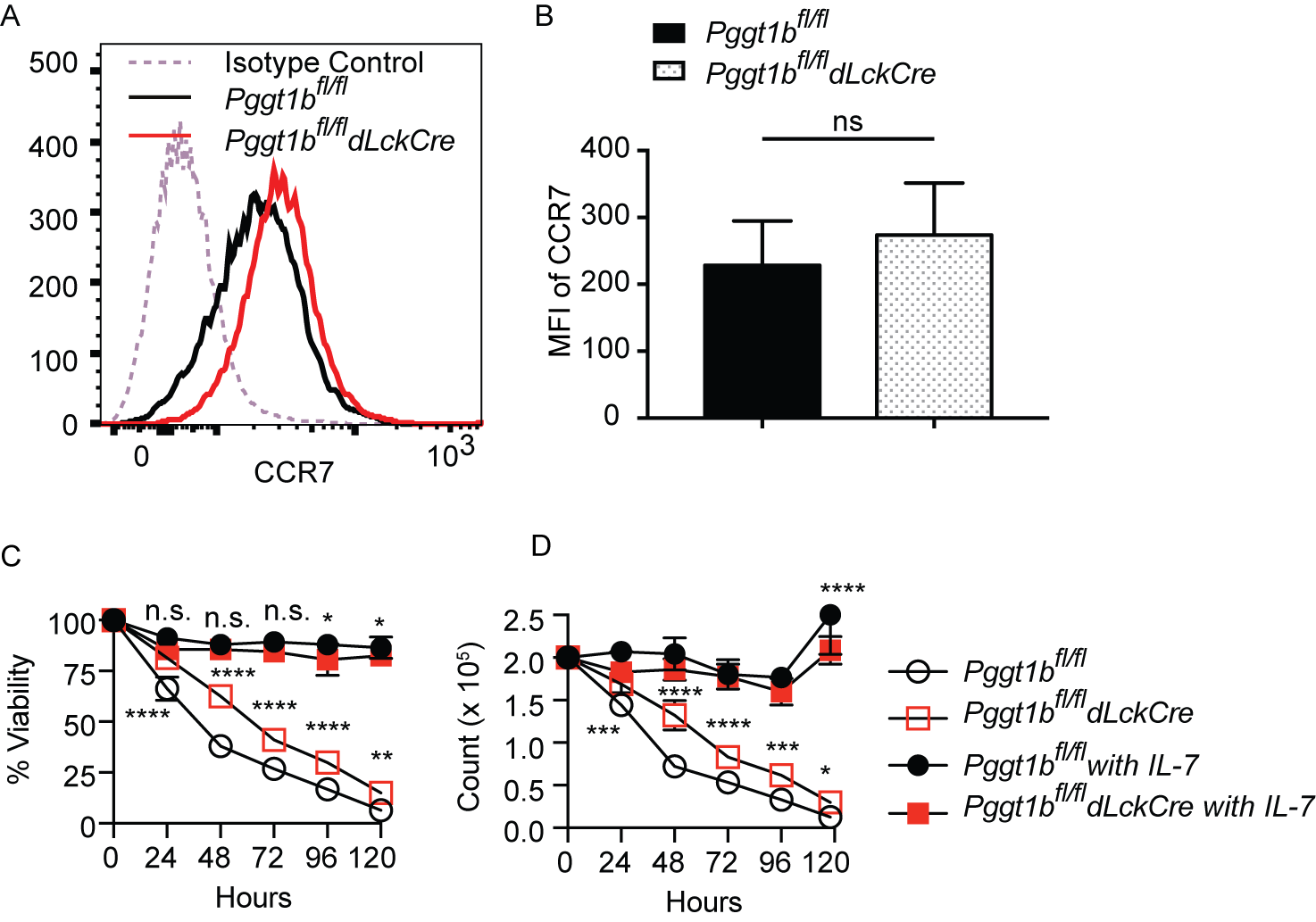

Supplement: Supplementary Figure 2 — CCR7 expression and in vitro survival of Pggt1b-deficient naive T cells. (A) Flow cytometry analysis of CCR7 expression on naive CD4+ T cells; (B) Mean fluorescence intensity of CCR7 described in (A); (C) percentage and (D) number of viable naive CD4+ T cells cultured in vitro in the absence or presence of mIL-7 (Results are representatives of at least two biologically independent experiments. n.s. statistically not significant; *p < 0.05, **p < 0.01, ***p < 0.001, ****p < 0.0001, unpaired t-test). [file Image_2.TIF]

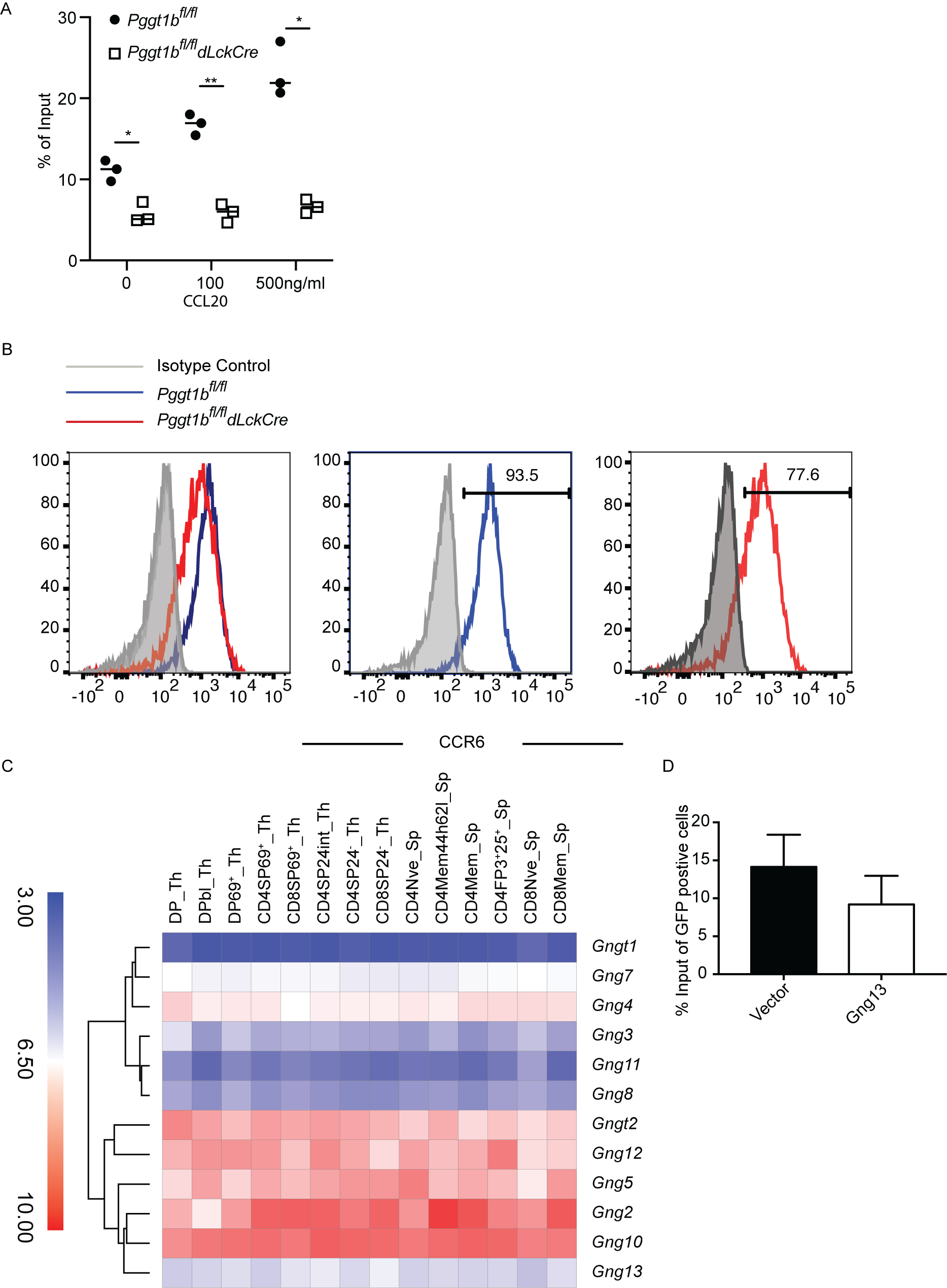

Supplement: Supplementary Figure 3 — Transmigration and CCR6 expression of Th17 cells and the expression of genes encoding the γ-subunits of heterotrimeric small GTPases in αβ T cells. (A) Percentage of input of Th17 cells transmigrated into the low chamber in response to CCL20 in transmigration assay; (B) Overlay of histogram of flow cytometry analysis of the expression of CCR6 on in vitro derived Th17 cells; (C) Array expression data were extracted from the Immgen consortium website and converted into logarithmic fold of changes and heatmap were generated using Morpheus web-based tools created by Broad Institute (https://software.broadinstitute.org/morpheus/); (D) percentage of input of GFP-positive, vector or Gng13-transduced Pggt1b-deficient Th17 cells transmigrated into the lower chamber in response to 500 ng/ml CCL20. [file Image_3.TIF]

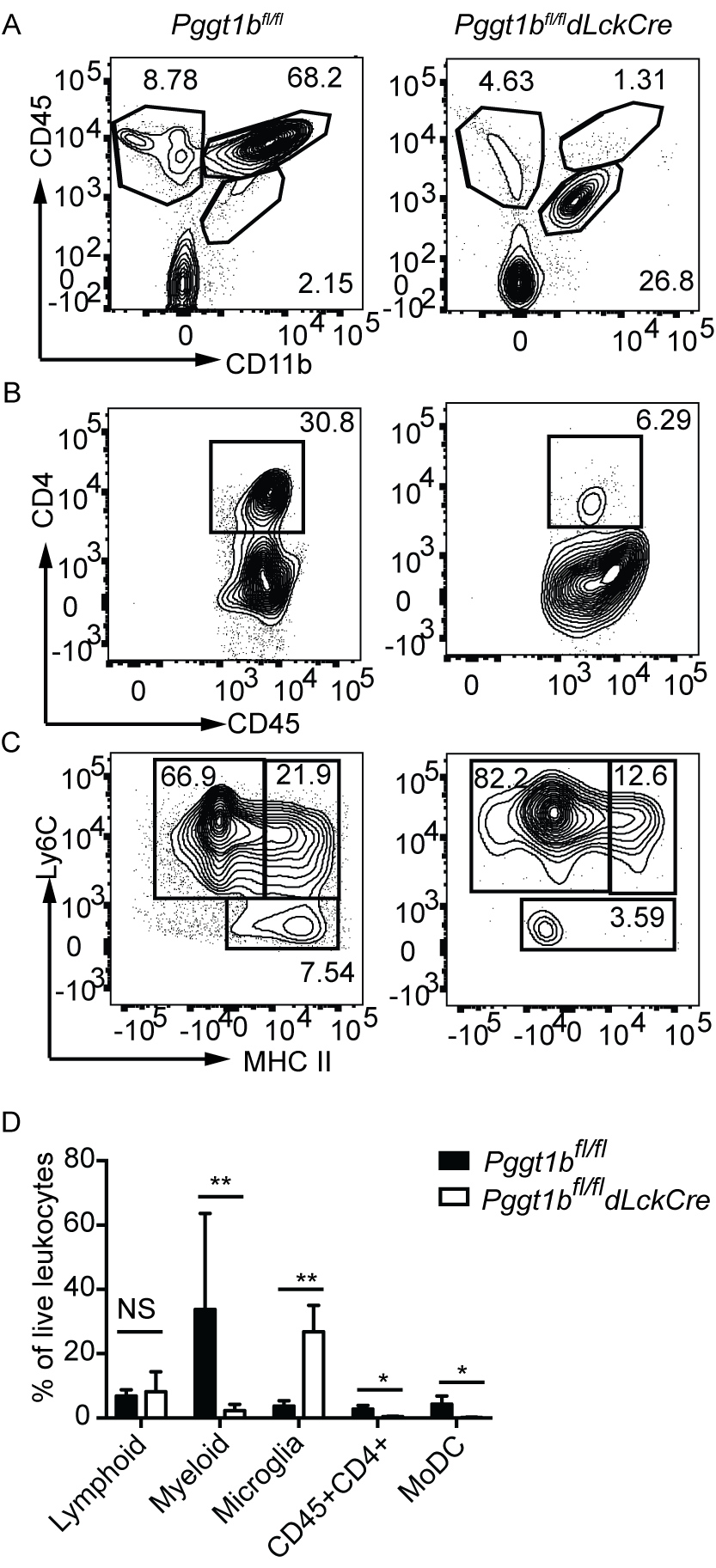

Supplement: Supplementary Figure 4 — Defective CD4 and monocyte-derived DC infiltration into the CNS of Pggt1bfl/fl dLckCre mice on day 14 after immunization. Mice were immunized as described in Figure 5, (A–C) Flow cytometry analysis of spinal cords leukocytes harvest on day 19 after immunization that were stained with antibodies against CD45, CD11b, CD4, Ly6C, Ly6G, CD44, CD64, and MHC II and gated according to a strategy described in the text to distinguish myeloid, lymphoid, microglia, CD4+ T cells, and monocyte-derived dendritic cells (MoDCs); (D) Percentage of lymphoid, myeloid, microglia, CD4+, and MoDCs in the spinal cord (Results are from two independent biological experiments with a total of 20 mice (10 male, 10 female) (NS, not significant, *p < 0.05, **p < 0.01, unpaired t-test). [file Image_4.TIF]

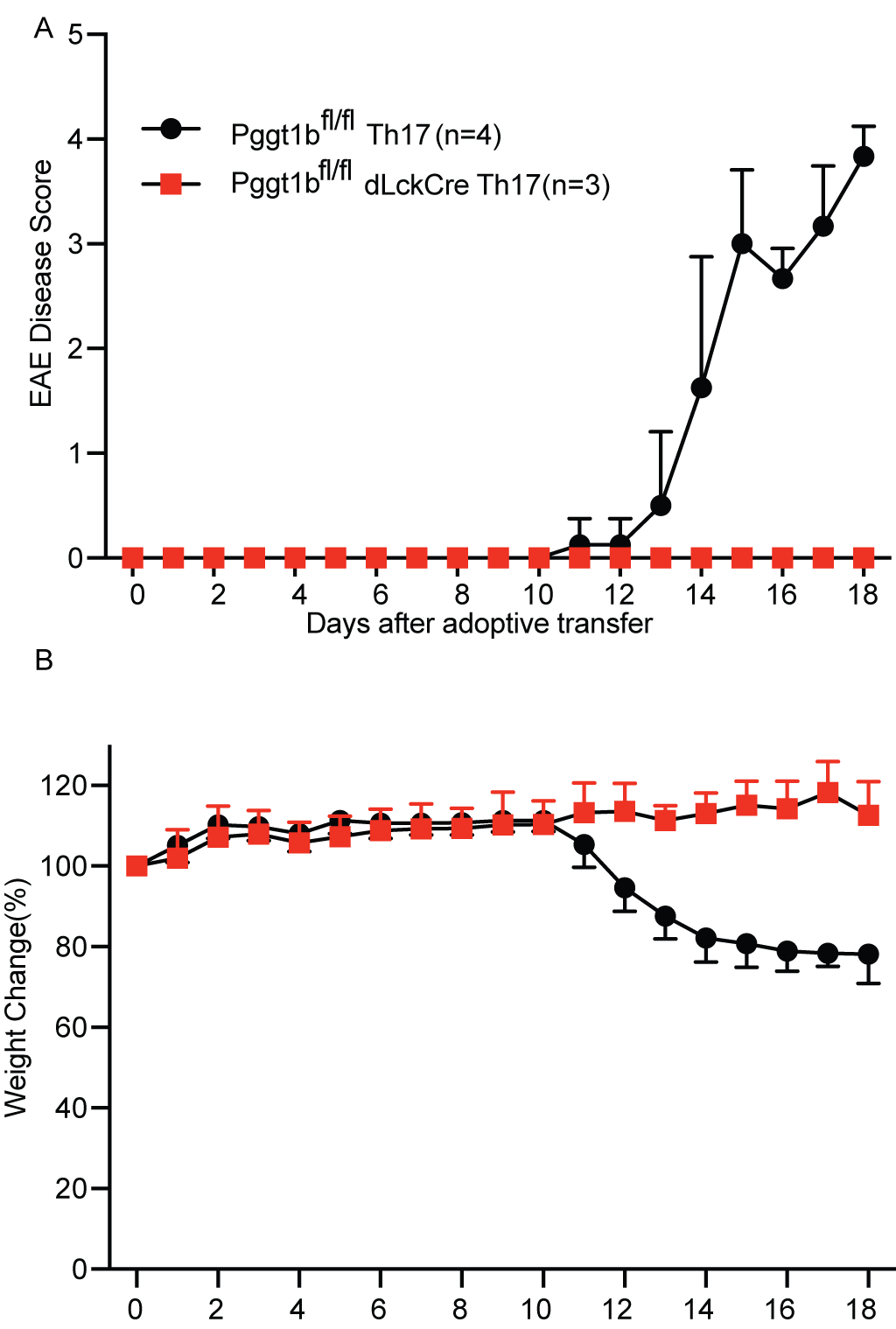

Supplement: Supplementary Figure 5 — Adoptively transferred 2D2-transgenic Pggt1b-deficient Th17 cells failed to induce EAE in recipient mice. (A) Clinical score of mice receiving in vitro differentiated inflammatory 2D2-transgenic Th17 cells; (B) Body weight change of mice described in (A). [file Image_5.TIF]

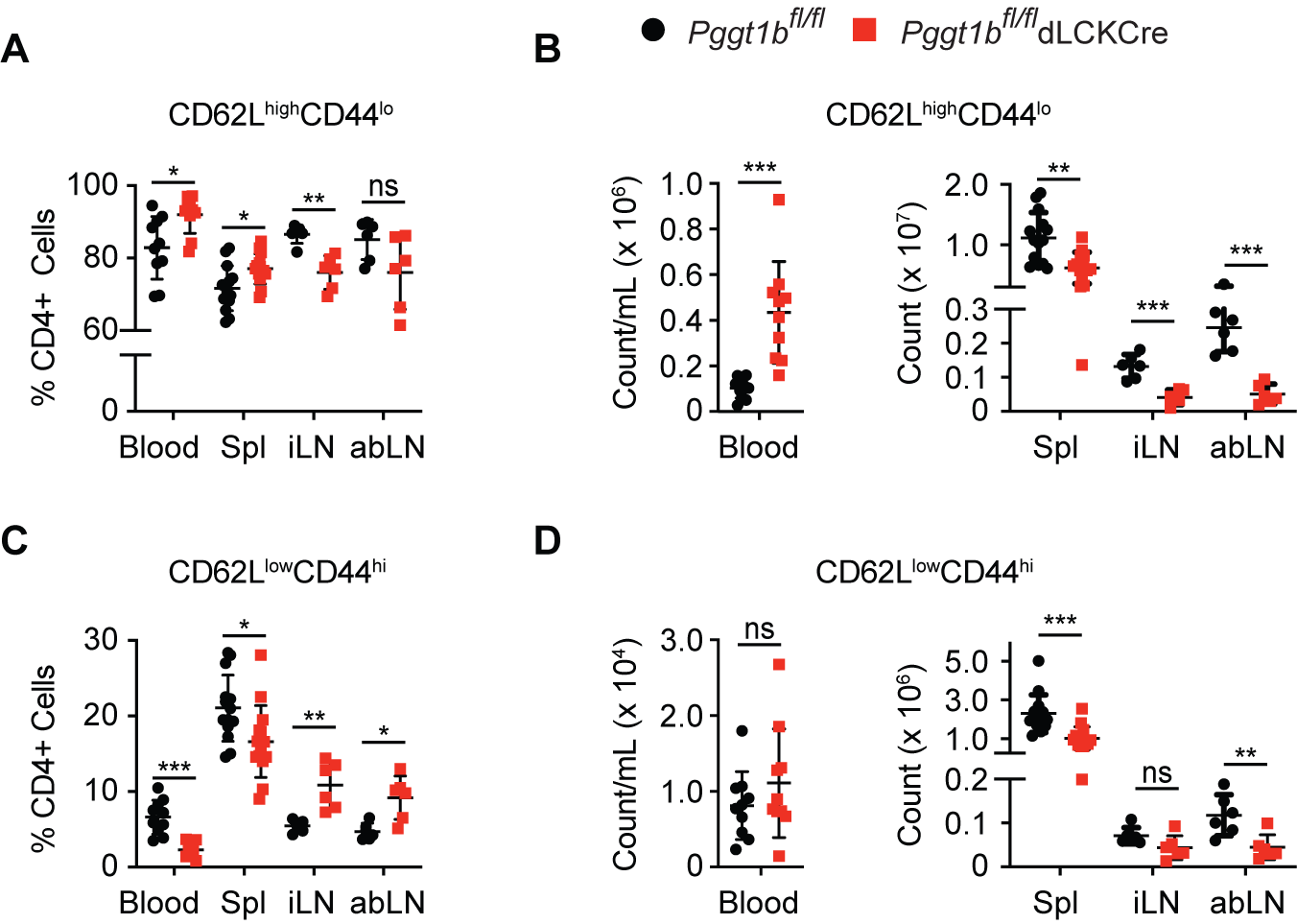

Supplement: Supplementary Figure 6 — Naive and effector/memory CD4+ T cells in the periphery. Percentage and number of naive (A,B) and effector/memory (C,D) CD4+ T cells in blood, spleen, inguinal (iLN), auxiliary and brachial (a/bLN) lymph nodes (Each dot represents an individual mouse, ns, not significant, *p < 0.05, **p < 0.01, ***p < 0.001, unpaired t-test). [file Image_6.TIF]

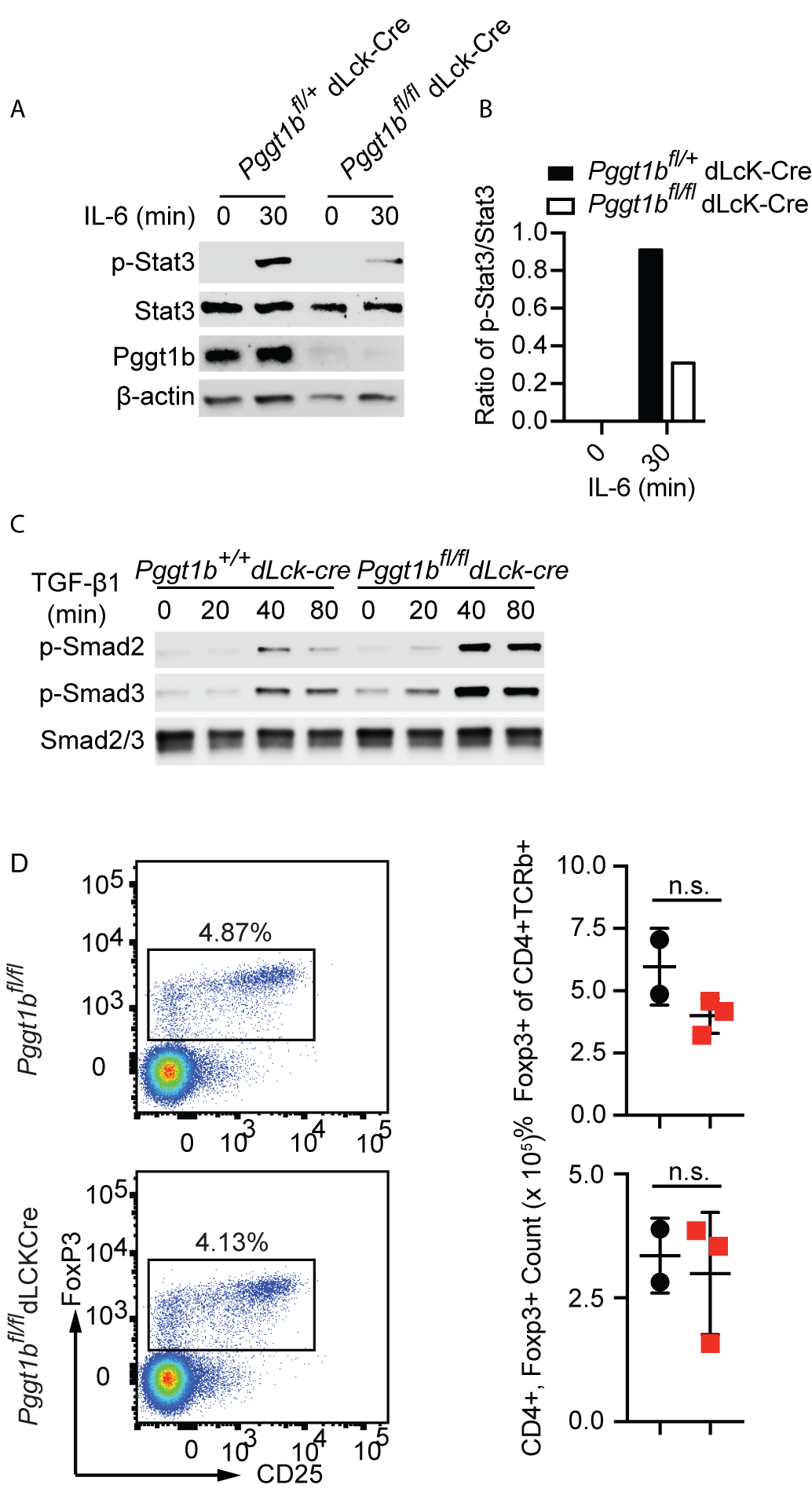

Supplement: Supplementary Figure 7 — Impaired IL-6-induced Stat3 phosphorylation and enhanced TGFβ1-induced Smad2 and Smad3 phosphorylation in Pggt1b-deficient naive CD4+ T cells. (A) Immuno-blot of naive CD4+ T cells stimulated with IL-6 with antibodies indicated in the figure; (B) Image J calculation of the phosphor-Stat3 as indicated in (A); (C) Western blot of phosphor-Smad2 and Smad3 of CD4+ naive T cells after TGFβ11 stimulation; (D) Flow cytometry analysis of thymic T regulatory cells. [file Image_7.TIF]
